# A ROS-responsive polymeric prodrug nanosystem with self-amplified drug release for PSMA (−) prostate cancer specific therapy

**DOI:** 10.1186/s12951-019-0521-z

**Published:** 2019-08-26

**Authors:** Yifan Wang, Yanqiu Zhang, Zhengxing Ru, Wei Song, Lin Chen, Hao Ma, Lizhu Sun

**Affiliations:** 1 Department of Oncology, Yancheng First People’s Hospital, Yancheng, 224005 China; 2 Department of Oncology, Shuyang Hospital Affiliated to Xuzhou Medical University, Shuyang People’s Hospital, Suqian, 223600 China; 3 Department of Oncology, Nanjing First Hospital, Affiliated to Nanjing Medical University, Nanjing, 210015 China; 4 Department of Oncology, Nanjing Hospital of T.C.M, Affiliated to Nanjing University of Traditional Chinese Medicine, Nanjing, 210001 China

**Keywords:** ROS-responsive, Self-accelerating, PMSA, Intracellular ROS amplification

## Abstract

**Background:**

The selectively accumulate in tumor site and completely release drug within cancer cells great limit the therapeutic effect of nano-drug delivery system. Moreover, absence of appropriate biomarker is one of the major challenges for prostate specific membrane antigen negative (PSMA (−)) prostate cancer therapy.

**Results:**

Herein, a PSMA (−) prostate cancer specific targeted and intracellular reactive oxygen species (ROS) amplification for ROS-responsive self-accelerating drug release nanoplatform (ATD-NPs) was developed. ATD-NPs was formed by three parts, including PSMA (−) prostate cancer specifically targeted part (DUP-PEG-DSPE), ROS-sensitive doxorubicin (DOX) polymeric prodrug (P(L-TK-DOX)), and the ROS generation agent (α-tocopheryl succinate, α-TOS); and this delivery system is expected to enhance PSMA (−) prostate cancer therapeutic effect, increase selective accumulation at tumor site and overcome intracellular incomplete drug release. After administration *i.v* injection, ATD-NPs could specifically accumulate in tumor site and markedly be internalized by cancer cells based on the DUP-1 (a PSMA (−) cancer cells specific target peptide). Subsequently, ATD-NPs could be dissociated under the high concentration reactive oxygen species (ROS) condition, resulting in DOX and α-TOS release. Then, the released α-TOS could be reacted with mitochondria to produce ROS, which in turn accelerating the release of drugs. Finally achieved the purpose of enhancing therapeutic efficacy and reducing side effect. Both in vitro and in vivo experiments demonstrated that the combination of tumor actively-targeted and self-amplifying ROS-responsive drug release showed more significant antitumor activity in the human PSMA (−) prostate cancer.

**Conclusion:**

The described technology unifies the tumor actively targets, self-amplified drug release, and excellent biocompatibility into one formulation, are promising for cancer treatment.

**Electronic supplementary material:**

The online version of this article (10.1186/s12951-019-0521-z) contains supplementary material, which is available to authorized users.

## Introduction

Prostate cancer is one of the major health concerns of male and is usually divided into prostate specific membrane antigen (PSMA) positive and negative two types according to the expression of PSMA [1]. PSMA is a type II integral membrane glycoprotein and overexpressed in prostate cells, and now it become a biomarker for prostate cancer diagnosis and a target for prostate cancer therapy [2, 3]. Up to now, many studies focused on PSMA (+) prostate cancer, but few studies of PSMA (−) prostate cancer have been carried out due to the absence of appropriate biomarker [4–8]. Therefore, rational designed drug delivery system which can target to PSMA (−) cancer cells may avail to prostate cancer therapy. Recently, Zitzmann et al. discovered a PSMA (−) ligand: DUP-1 peptide, which could specifically target to PSMA (−) cells such as PC-3 cells and DU145 cells [7–9]. Therefore, DUP-1 could be employed as a ligand for PSMA (−) tumor actively-targeted therapy.

Recently, polymeric prodrug micellular-based drug delivery nanosystem (PPM-DDS) has received the favor by researchers [10–12]. In which, the drug was conjugated to biocompatible polymers to form a polymeric prodrug, subsequently, the prodrug would translate into active drug once exposed to tumor microenvironment [13, 14]. As compared with traditional drug loading methods through non-covalent interactions, the PPM-DDS could achieve high drug loading efficiency, prolong drug circulation time, avoid premature release, reduce side effects and enhance therapy efficacy [15, 16]. However, only a few PPM-DDS have achieved good results both in vitro and in vivo, because there are many drawbacks still unsolved and hinder the clinical application of PPM-DDS, specifically including specific target to tumor cells, poor cellular uptake and incomplete drug release [17–19]. To improve delivery efficiency and specificity tumor-targeting of PPM-DDS, ligand-mediated tumor actively-targeted may be a good strategy. Because after modification targeting ligand on the surface of nanomedicine not only can increase tumor site accumulation, but also can promote cellular uptake [20–23]. Various targeted ligands have been developed for targeted drug delivery, such as antibodies, peptides, etc. [24–28].

Moreover, after internalized by cancer cells, the PPM-DDS should rapidly and completely release drug under a tumor-specific stimulation to exert drugs high therapeutic efficiency to tumors [15, 19, 29]. Previous studies have been found that the concentration of reactive oxygen species [ROS, including superoxides (O_2_^−^), hydroxyl radicals (OH·), and hydrogen peroxides (H_2_O_2_)] in cancer cells was obviously higher than that of normal cells [30–33]. Hence, PPM-DDS with ROS-sensitive drug release characteristic is a promising approach to achieve selective and rapid drug release in tumor cells. To construct ROS-responsive drug delivery system, various oxidation-labile groups such as boronic ester, alkylene, and thioketal (TK) have been investigated to develop drug delivery system for tumor treatment [34–36]. However, affected by the tumor heterogeneity, very few of aforementioned ROS-responsive materials show sufficient sensitivity to efficiently control drug release in cancer cells, because the endogenous ROS levels are too low to trigger drug release [15, 28, 29, 37]. Thus, tumor active-targeted ROS-responsive polymeric prodrug delivery system with ROS generation ability will be a good strategy to promote drug release in cancer cells.

**Scheme 1:**
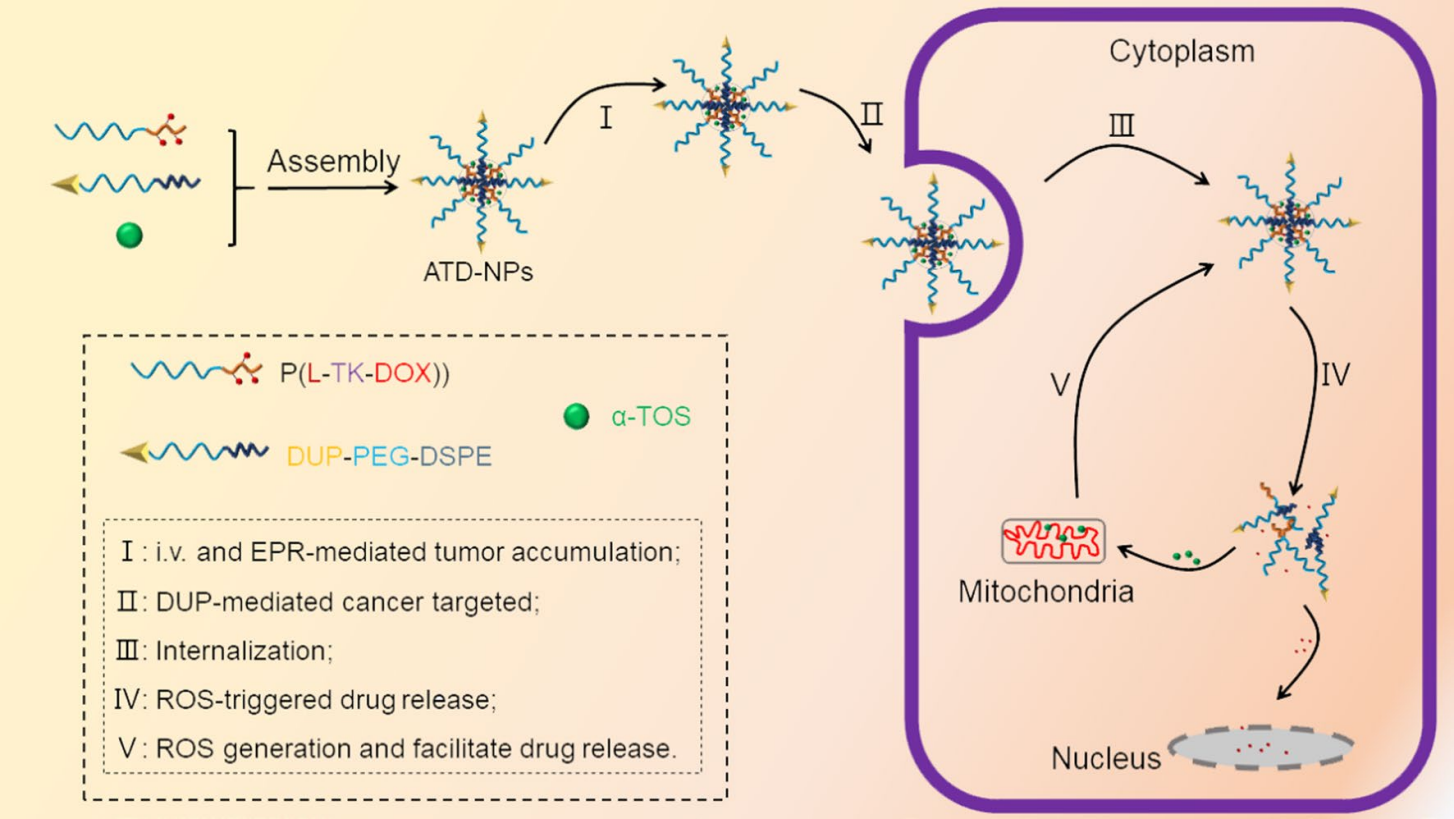
Schematic illustration of ATD-NPs for PSMA (−) prostate cancer-specific targeted and self-amplifiable drug release

To overcome the aforementioned concerns, here, we proposed a ROS-responsive cascade amplification drug release polymeric prodrug nanoplatform (defined as ATD-NPs) for PSMA (−) prostate tumor active targeted therapy (Scheme 1). Firstly, a ROS-response polymeric prodrug was developed by conjugating DOX to the side of methoxy poly(ethylene glycol)-*b*-poly(l-lysine) (PEG-*b*-PLL) copolymer through a ROS-sensitive linker (thioketal linker, TK). Then, the PSMA (−) prostate cancer cells specifically targeting module was produced by conjugating DUP-1 peptide to DSPE-PEG (DUP-PEG-DSPE). Finally, the ROS generation agent (α-tocopheryl succinate, α-TOS), ROS-sensitive polymer prodrug, and tumor actively-targeted three parts self-assembled in aqueous solution to form micelles (ATD-NPs). After tail vein administration, ATD-NPs nanoparticles accumulated in tumor tissue through the enhanced permeability and retention (EPR) effect and DUP-1 mediated actively tumor targeting; and then, the ATD-NPs was internalized by PSMA (−) cancer cells. Subsequently, the high concentration ROS would trigger DOX from ATD-NPs by breaking the H_2_O_2_-sensitive TK linker, this resulting in disassembly of ATD-NPs and the release of α-TOS. Moreover, the released α-TOS could interact with mitochondria and produce ROS, in turn amplify the micelle disassembly and drug release, and thus circulating. We hope the combination of tumor actively-targeted and intracellular ROS amplification in one ATD-NPs would reduce the side effects of the encapsulated anticancer drug and enhance therapeutic efficacy.

## Experiment section

### Preparation α-TOS and DOX co-loaded active targeted micelles (ATD-NPs)

The α-TOS and DOX co-loaded active tumor targeting micelles (ATD-NPs) were prepared by coprecipitation method [32]. Typically, 800 µg of P(L-TK-DOX), 100 µg DUP-PEG-DSPE, and 110 µg of α-TOS were dissolved in 200 µL of DMF and stirred at room temperature for 1 h. After that, the mixture was added into 2 mL of double distilled water drop by drop under vigorously stirring over for 2 h, and then the mixture was transferred to a dialysis bag (MWCO = 3500 Da) dialyzed against distill water for 6 h at 4 °C under dark to remove DMF. The final ATD-NPs was obtained after removal of large particles and unloaded α-TOS using a 200 nm aperture filter. The control groups, AD-NPs and TD-NPs were also prepared by the same method (Additional file 1: Table S1).

The drug loading efficiency (DLC) and drug encapsulation efficiency (DEE) of α-TOS was measured by a high-performance liquid chromatography (HPLC) method as reported previous [38]. The HPLC analysis was performed using a phase column (Agilent ODS C18 column, 4.6 × 250 mm^2^, 5 μm particle size) eluted with acetonitrile:methanol with 0.04% trifluoroacetic acid (60/40, v/v) at a flow rate of 1.5 mL/min and the column effluent was monitored by UV detector set at 280 nm. And the DLC and DEE were calculated according to the following formula:

$$ {\text{DLC}}\,\left( {{\text{wt}}\% } \right) = \frac{\text{weight of drug in the micelles}}{\text{weight of the whole micelles}} \times 100\% $$

$$ {\text{DEE}}\,\left( {{\text{wt}}\% } \right) = \frac{\text{weight of drug in the micelles}}{\text{weight of feed drug}} \times 100\% $$

### Intracellular ROS productions and associated mechanisms

The concentration of intracellular ROS was detected by fluorescence microscope and flow cytometer by using dichlorofluorescin diacetate (DCFA-DA) as a probe. For fluorescence microscope assay, PC-3 cells were seeded in the six-well plates at a density of 1 × 10^4^ cells per well for 48 h. The cells were incubated with free α-TOS for different concentration or different incubation times. Cells without any treatment were used as a control. After treatment, the cells were washed with pre-cold PBS for three times and the media were replaced with DCFH-DA at 37 °C for 20 min. After washed, the cells were fixed by 4% paraformaldehyde and then observed by an inverted fluorescence microscope. Moreover, the PC-3 cells treated with α-TOS, AD-NPs, TD-NPs, and ATD-NPs for 4 h (equivalent to DOX 5 µg/mL or α-TOS 2.5 µg/mL), respectively, were also observed by fluorescence microscope.

For flow cytometer quantitative analysis, PC-3 cells were treated with α-TOS, AD-NPs, TD-NPs, or ATD-NPs for different incubation time or different α-TOS concentration at same incubation time. After that, intracellular ROS was stained by DCFH-DA. Then, the cells were collected, washed with PBS and quickly measured by flow cytometer. For analysis of dose-dependent ROS production, PC-3 cells were treated with α-TOS or prodrug nanoparticles for 4 h at equivalent α-TOS dose ranging from 0.5 to 16 µg/mL. To evaluate the time-dependent ROS generation, PC-3 cells were incubated with α-TOS or prodrug micelles for different times at equivalent to 10 µg/mL of α-TOS. Moreover, the concentration- and time-dependent ROS changes in PMSA (+) LNCaP cells were also measured after treated with α-TOS or prodrug micelles.

It is reported that the α-TOS was reacted with mitochondrial respiratory complex II to produce ROS in cancer cells. Therefore, the activity of mitochondrial respiratory complex II was detected using a Mitochondrial Complex II Activity Assay Kit. Typically, PC-3 cells and LNCaP cells were seeded into 12-well plates at a density of 1 × 10^4^ cells/well and incubated for 48 h. After that, the cells were treated with α-TOS, ATD-NPs, TD-NPs or AD-NPs at equivalent α-TOS concentration of 10 µg/mL. At interval times point, cells were lysed, and the cell protein content was determined by BCA Protein Assay Kit, as well as the activity of mitochondrial respiratory complex II was detected by Mitochondrial Complex II Activity Assay Kit. The relative activity of mitochondrial respiratory complex II was calculated by the followed formula:

$$ {\text{Relative activity}}\,\left( \% \right) = \frac{Ae}{Ac} \times 100\% $$

where *Ae* is the activity of mitochondrial respiratory complex II at different times and *Ac* is the mitochondrial respiratory complex II of negative control.

### Intracellular drug release

Intracellular ROS-responsive drug release of ATD-NPs were investigated by confocal scanning laser microscope (CLSM, ZEISS LSM700) and HPLC. For CLSM assay, the PC-3 cells were seeded on laser confocal small dish at the density of 1 × 10^4^ and incubated for 48 h. Then, the cells were treated with ATD-NPs, TD-NPs, AD-NPs or AD-NPs + α-TOS for 12 h with the final DOX concentration of 5 µg/mL. After incubation, cells were fixed by 4% paraformaldehyde, stained by DAPI, and then observed by CLSM. The excitation and emission wavelength of DOX was 488 nm and 552 nm, respectively. The excitation and emission wavelength of DAPI was 364 nm and 454 nm, respectively.

For the HPLC study, PC-3 cells and LNCaP cells were seeded on six-well plates and incubated for 48 h. Then, cells were treated with ATD-NPs, TD-NPs, AD-NPs or AD-NPs + α-TOS for 8 h, 12 h, 24 h, or 36 h. After incubation, the cells were washed twice with cold PBS. Subsequently, 200 μL of cell lysis buffer (1% of TritonX-100) was added and incubated for 30 min. Then, the cell lysate (100 μL) was mixed with acetonitrile (200 μL) by ultrasonication for drug extraction followed by centrifugation at 8000 rpm for 10 min, the supernatant was collected and the concentration of active DOX was measured by HPLC [10]. All the determination of DOX content was normalized to protein concentrations of cell lysate. The protein concentration of cells was measured by BCA kit.

### In vivo imaging of mouse with xenograft tumor

The prostate cancer tumor model was established by subcutaneous injection of 7 × 10^6^ PC-3 cells into the right side back of male nude mice. After 2 weeks, the Cy5.5 loaded ATD-NPs or TD-NPs nanoparticles was intravenously injected via the tail vein. At 12, 24, 36, and 48 h post injection, the mice were imaged on IVIS Lumina imaging system (Caliper, USA). Thereafter, the mice were euthanized at 48 h post injection, tumors and the major organs, such as heart, live, spleen, lung, and kidney, and subjected to ex vivo fluorescence imaging.

### Pharmacokinetic and biodistribution studies

For pharmacokinetic assay, ICR mice were randomly divided into two groups (n = 3 per group) and then intravenously injected with DOX, TD-NPs, or ATD-NPs at a DOX-equivalent dose of 5 mg/kg. At the predetermined times, blood samples were collected, and centrifuged at 6000 rpm at 4 °C for 10 min, and then 20 µL of the supernatant plasma was mixed with 80 µL of acetonitrile to precipitate all the proteins. After centrifugation, the supernatant was collected and concentrated, and subsequently, the concentration of DOX were determined using a FLX800 TB microplate reader (BioTek, USA) with fluorescence excitation at 485 nm and emission at 590 nm. The background plasma fluorescence was eliminated through three untreated mice’s plasma.

For biodistribution study, PC-3 xenografted tumor mice were treated with DOX, TD-NPs, or ATD-NPs at a DOX-equivalent dose of 5 mg/kg, respectively. At 4, 12 and 24 h post injection, mice were sacrificed and heart, liver, spleen, lung, kidney, and tumor were dissected, weighed, and homogenized, centrifuged and collected the supernatants. Subsequently, the DOX concentration were detected according to abovementioned. The background tissue fluorescence was eliminated three untreated mice’s tissue.

### In vivo antitumor effects

Mice bearing PC-3 tumors were randomly divided into five groups (*n* = 6) and intravenously injected with saline, DOX, AD-NPs, TD-NPs, or ATD-NPs at an equivalent DOX injection dose of 5 mg/kg. The treatment was implemented by *i.v.* injection every 3 days 4 times [31]. The body weight and the tumor volumes were measured at intervals of 3 days. Tumor volumes were calculated by the formula:

$$ {\text{Volume}} = ({\text{L}} \times {\text{W}}^{2} )/2, $$

where L and W are the largest and smallest diameters of tumor, respectively. After 21 days, the mice were sacrificed; the major organs or tissues including heart, liver, spleen, lung, kidney, and tumor tissues were collected and fixed in 4% formaldehyde for histological examination. Hematoxylin and eosin (H&E) staining was taken to evaluate the acute toxicity.

### Statistical analysis

All the results were expressed as mean ± standard deviation (SD). The differences among groups were calculated using Student’s t-test or one-way ANOVA analysis. Differences were considered significant when *p < 0.05, **p < 0.001, ***p < 0.0001, respectively.

## Results and discussion

### Preparation and characterization of synthesized polymers

**Fig. 1:**
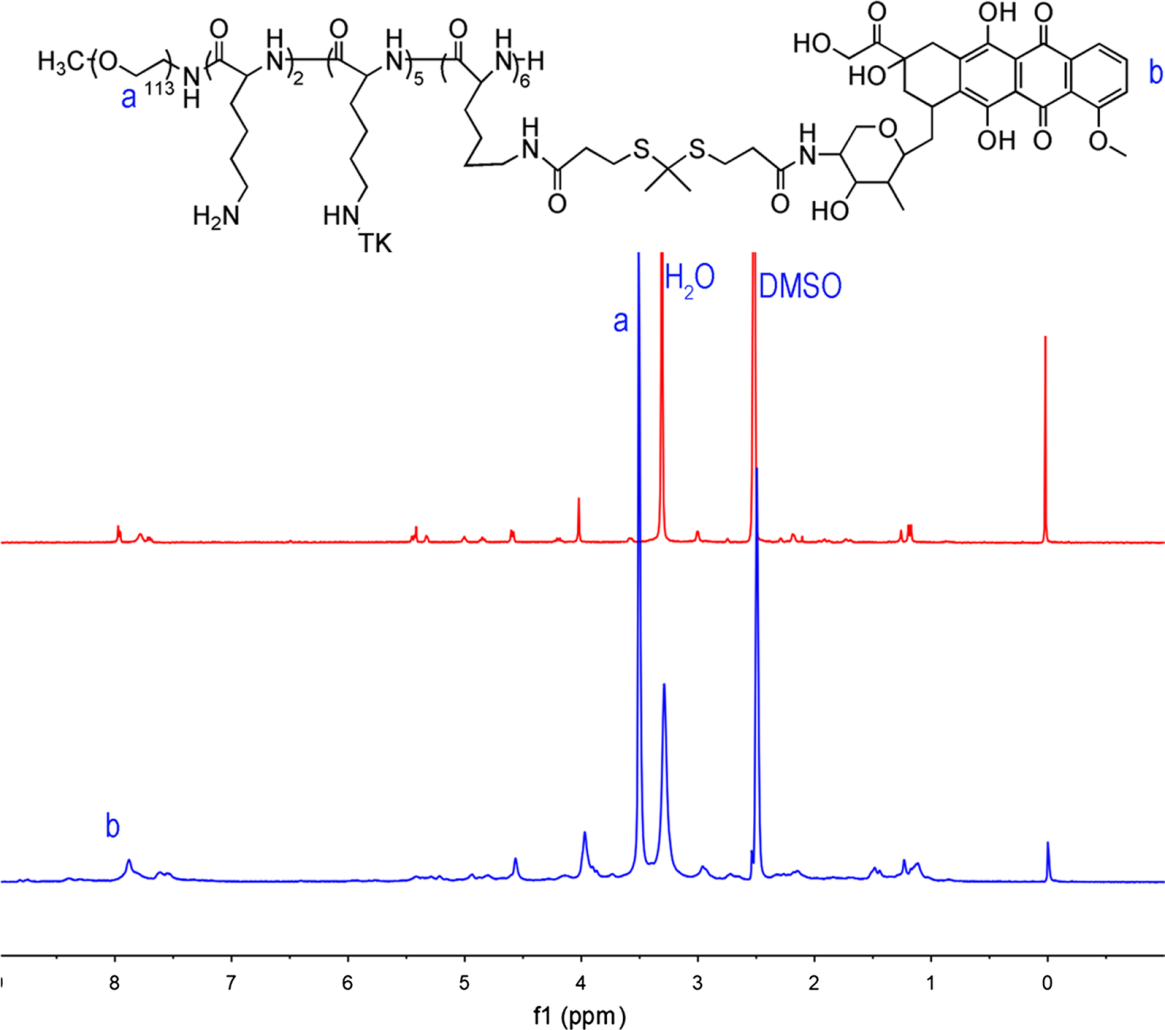
^1^H NMR spectrum of DOX and P(L-TK-DOX) in DMSO-*d6*

The synthesis route of P(L-TK-DOX) was shown in Additional file 1: Scheme S1A. Firstly, the ROS-sensitive linker, TK, was synthesized according to previous reports [35], and its chemical structure was characterized by ^1^H NMR. As shown in Additional file 1: Figs. S1 and S2, both ^1^H NMR spectrum and mass spectrum demonstrated that the TK was successfully synthesized and this result was consistent with previous report [14, 36]. Secondly, PEG-*b*-PLL was synthesized by ring opening polymerization and removed the benzyl group under acid condition (Additional file 1: Scheme S1C). The products were also characterized by ^1^H NMR. As shown in Additional file 1: Fig. S3, in the spectrum of PEG-*b*-PLLZ, the peaks were consistent with the previous reports [10, 11, 39, 40]. The signal at *δ* 2.8 ppm were contributed to the PEG; the signal at *δ* 2.8 ppm was assigned to the lysine; the signals at *δ* 7.1–7.8 ppm were belong to the benzyl group of PLLZ. In the PEG-*b*-PLL spectra, the signal at *δ* 4.9 and 7.1–7.4 ppm disappeared, demonstrated that the benzyl group of PLLZ was deprotected completely. The degree of polymerization (DP) of PEG-*b*-PLLZ and PEG-*b*-PLL was calculated by comparing the signal intensities of lysine methylene protons with methylene protons of PEG according to previous reports [11, 12], and both the value was 15. In addition, GPC analyses demonstrated that both PEG-*b*-PLLZ and PEG-*b*-PLL had a narrow molecular weight distribution and lysine DP was about 15 (Table 1). Finally, DOX and TK was conjugated to the side chain of PEG-*b*-PLL to obtain the ROS-sensitive polymer prodrug, P(L-TK-DOX) (Additional file 1: Scheme S1C). The ^1^H NMR spectrum of PEG-b-P(LL-g-TK) was shown in Additional file 1: Fig. S4, the signal at *δ* 2.8 ppm belonged to TK, this suggested that TK was successfully conjugated to PEG-*b*-PLL. As exhibited in Fig. 1, the typical signals of phenyl proton (7.0–8.0 ppm) of DOX appeared in the P(L-TK-DOX) spectrum, demonstrated that DOX was conjugated to PEG-*b*-P(LL-*g*-TK). Moreover, the DP of TK and DOX calculated according to ^1^H NMR and GPC were 13 and 6, respectively (Table 1). These results demonstrated that the ROS-sensitive polymer prodrug was successfully synthesized. At the same time, DUP-PEG-DSPE was prepared by Michael addition reaction between DUP-1 and Mal-PEG-DSPE (Additional file 1: Scheme S1B), and the structure of Mal-PEG-DSPE and DUP-PEG-DSPE was confirmed by ^1^H NMR. As shown in Additional file 1: Fig. S5, the characteristic peak of maleimide at *δ* 6.7 ppm in Mal-PEG-DPSE, and it disappeared in DUP-PEG-DSPE as well as the peaks of DUP-1 appeared in DUP-PEG-DSPE, demonstrated that the DUP-PEG-DSPE was synthesized successfully.

**Table 1:** Characterization of the copolymers

Copolymer	Composition ratio^a^	Mn^a^ (Da)	Mn^b^ (Da)	PDI^b^
PEG-*b*-PLLZ	113:15	9315	9166	1.08
PEG-*b*-PLL	113:15	6874	6953	1.07
PEG-*b*-P(LL-g-TK)	113:15:13	10,178	10,273	1.13
P(L-TK-DOX)	113:15:13:6	13,568	13,947	1.16

^a^Estimated by ^1^H NMR

^b^Detected by GPC

### Characterization of ATD-NPs

**Fig. 2:**
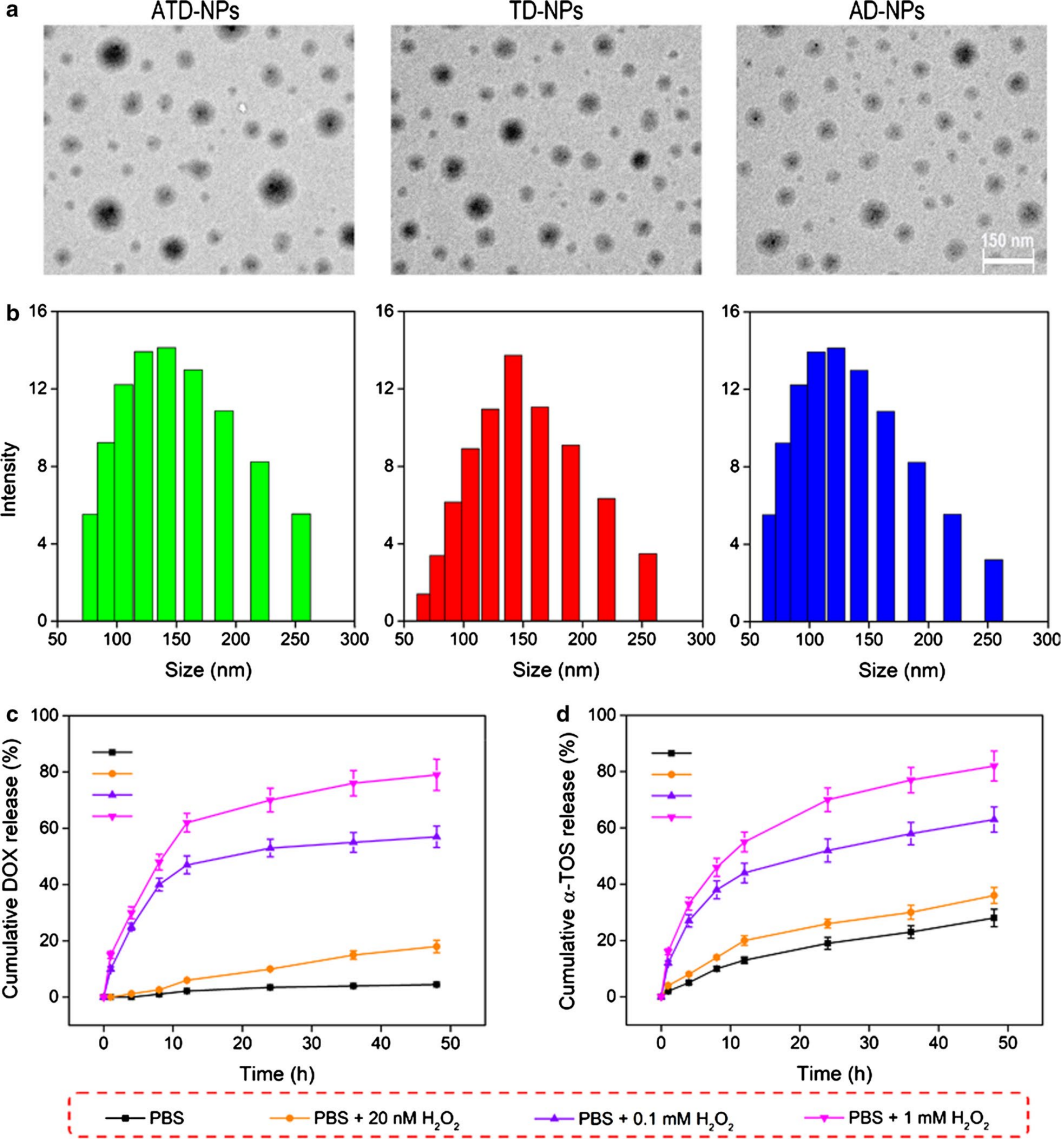
**a**, **b** TEM image (**a**) and DLS image (**b**) of ATD-NPs, TD-NPs, and AD-NPs. **c**, **d** DOX (**c**) and α-TOS (**d**) released form ATD-NPs in presence different ROS environments. Data showed ± SD, *n* = 3

The α-TOS and DOX co-loaded targeted micelles (abbreviated as ATD-NPs); the α-TOS and DOX co-loaded non-targeted micelles (abbreviated as TD-NPs); and the single DOX loaded targeted micelles (abbreviated as AD-NPs) were prepared by a precipitation method. The component of three micelles were exhibited in Additional file 1: Table S1. The TEM images and size range histograms of prodrug micelles were shown in Fig. 2a, b, and their main properties were exhibited in Table 2. It can be observed that the three micelles showed a compact and spherical morphologies with uniform size distribution and moderate polydispersity index (PDI < 0.3). Moreover, the zeta potential of ATD-NPs, TD-NPs, and AD-NPs were − 27.2, − 13.5 and − 24.7 mV, respectively (Table 2). Additionally, all prodrug micelles have a relatively low CMC values (< 20 µg/mL) as illustrated in Additional file 1: Fig. S6. The slightly negative surface charges with low CMC will contribute to the better blood compatibility and prolong circulation time of nanomedicines due to reduced interactions with blood components and maintain stable in bloodstream [5, 12]. To demonstrate the stability of prodrug micelles, all the micelles were incubated with PBS or PBS content 10% fetal bovine serum (FBS) at 37 °C for 48 h, respectively. The results were shown in Additional file 1: Fig. S7A and B, we observed no obvious change in particle size of ATD-NPs, TD-NPs, and AD-NPs, indicating that these micelles with PEG shells were stable in the presence of serum. Furthermore, the hemolytic of the three micelles were evaluated by hemolysis assay, the result was shown in Additional file 1: Fig. S8, no significant hemolytic of all micelles was observed, and the hemolysis rate of all micelles were lower than 5% even the concentration of micelles reach up to 5 mg/mL. These results demonstrated that the three micelles have an excellent biocompatibility and physiological stability potential. In addition, the drug loading content for DOX in the ATD-NPs, TD-NPs, and AD-NPs reached up to 19.7%, 20.8% and 22.4%, respectively (Table 2).

**Table 2:** Characterization of nanoparticles

Nanoparticles	Size (nm)	PDI	Zeta (mV)	DLC of DOX (%)	DLC of α-TOS (%)	DEE of α-TOS (%)
ATD-NPs	141	0.27	− 27.2	19.7	9.4	91.2
TD-NPs	106	0.23	− 13.5	20.8	9.8	93.5
AD-NPs	125	0.28	− 24.7	22.4	–	–

### ROS-triggered drug release

After exposed to high level ROS in cancers, the TK linker between DOX and polymer can cleave and release DOX and α-TOS. To study the responsive drug release behavior, H_2_O_2_ was used as a ROS stimulus, and the release of DOX and α-TOS from ATD-NPs at different concentration of H_2_O_2_ were detected by HPLC. As exhibited in Fig. 2c, under no ROS condition, less than 5% of DOX were released from the ATD-NPs even incubated for 48 h, which is beneficial to avoid the side effect of DOX. As expect, only about 18% of DOX were released at low ROS level (20 nM of H_2_O_2_), however, when the concentration of H_2_O_2_ increased to 0.1 mM and 1 mM, the accumulative release of DOX increased to 57% and 79% after incubated for 48 h. Moreover, as shown in Fig. 2d, α-TOS was released more quickly than that of DOX both with or without ROS conditions, this may attribute to the non-covalent interactions between α-TOS and polymers. Additionally, with the increase of ROS concentration, the total accumulation release of α-TOS increased. This may be attributed to the break of TK, which resulted in disassembly of ATD-NPs, and then led to α-TOS accelerated release. The release profiles of both DOX and α-TOS were ROS concentration-dependent, demonstrated that the ATD-NPs has a good ROS-sensitive drug release capacity. Apparently, DOX released from ATD-NPs depended on the concentration of H_2_O_2_, which was the basement of our self-accelerating drug release in cellular environment.

### DUP-1 mediated active tumor targeting ability

**Fig. 3:**
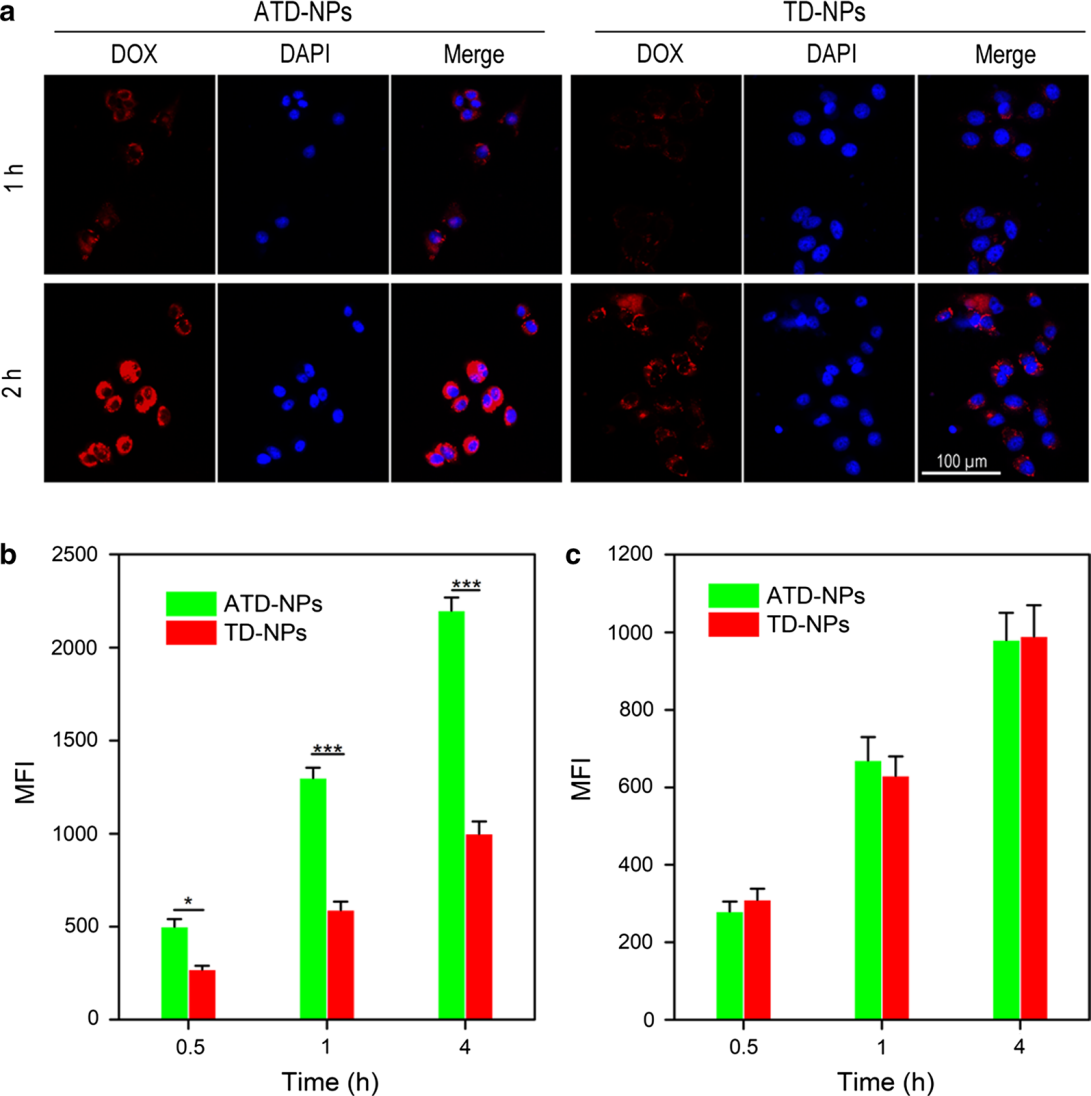
**a** CLSM images of PC-3 cells treated with ATD-NPs or TD-NPs for 1 or 2 h. **b**, **c** Quantitatively analysis of cellular uptake of PC-3 cells (**b**) and LNCaP cells after treated with TD-NPs and ATD-NPs, respectively. Data showed mean ± SD, *n* = 3

To confirm the selective cell uptake of the ATD-NPs, the cellular uptake of PSMA (−) prostate cancer PC-3 cells for targeted ATD-NPs micelles and non-targeted TD-NPs micelles were observed by confocal laser scanning microscopy (CLSM). As shown in Fig. 3a, the fluorescence intensity increased with increasing incubation time at both groups. The red fluorescence signal of DOX in ATD-NPs treated group was stronger than TD-NPs group either incubated for 1 h or 2 h. Moreover, selectively cellular uptake was also quantitatively analyzed both in PSMA (+) LNCaP cells and PSMA (−) PC-3 cells. As exhibited in Fig. 3b, the mean fluorescence intensity (MFI) of DOX in PC-3 cells treated with ATD-NPs group was 1.2-, 1.8-, 2.7-, and 3.2-fold than that of TD-NPs group after incubated for 0.5 h, 1 h, 2 h, and 4 h, respectively. However, no significant difference of MFI can be observed in LNCaP cells after incubated ATD-NPs or TD-NPs for different time (Fig. 3c). Taken together, these observations clearly indicated that ATD-NPs micelles could be selectively uptake by PSMA (−) cells originating from the targeting ability of DUP-1 peptide.

### ROS generation ability of α-TOS in cells and associated mechanisms

It is reported that α-tocopheryl succinate (α-TOS), a vitamin E analogue, which could rapidly generate ROS in cells after interacting with mitochondrial respiratory complex II and interfering the electron transportation chain in mitochondria [41–43]. To study this phenomenon, we had examined the efficiency of α-TOS-induced ROS generation in human prostate cancer PC-3 and LNCaP cells. The prevailing intracellular ROS sensitive probe 2′,7-dichlorofluorescein diacetate (DCFH-DA) was utilized to detect the ROS generation, which could be rapidly oxidized to dichlorofluorescein (DCF) with green fluorescence by the intracellular ROS [44].

**Fig. 4:**
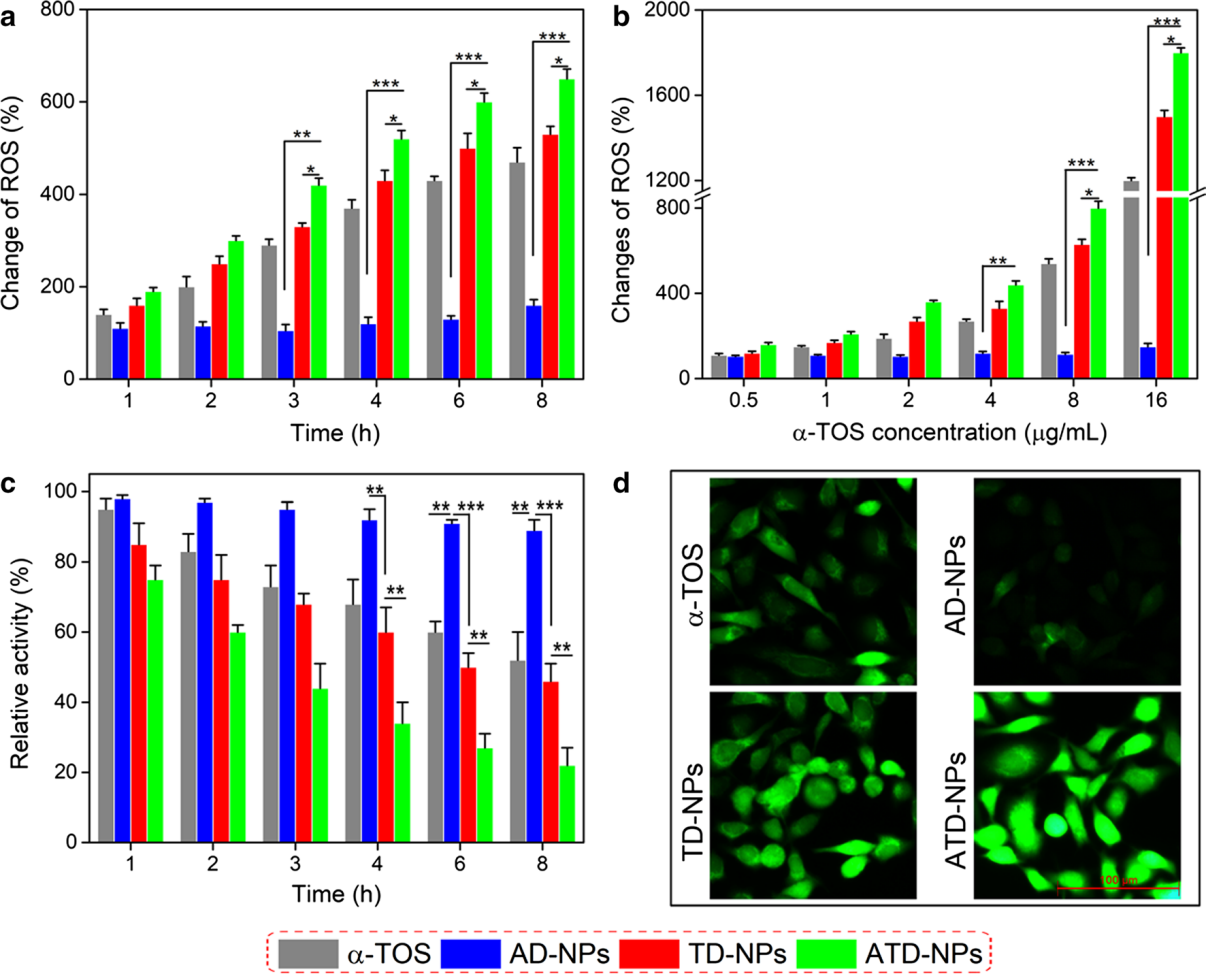
ROS changes in PC-3 cells after treated with α-TOS, TD-NPs, AD-NPs, and ATD-NPs for different incubation times (**a**) and different concentration (**b**). For **a**, the concentration of α-TOS in all group was fixed at 6 µg/mL; for **b**, all group was treated for 4 h. **c** The relative activity of mitochondrial respiratory complex II in PC-3 cells after incubated with α-TOS, TD-NPs, AD-NPs, and ATD-NPs for different time. **d** Fluorescence microscope images of intracellular ROS production in PC-3 cells after treated for 4 h. All data showed mean ± SD, *n* = 3. *p < 0.05, **p < 0.01, ***p < 0.001

Fluorescence microscope assay demonstrated that the capacity of α-TOS generation ROS was both dose- and time-dependent in PC-3 cells (Additional file 1: Fig. S9). Furthermore, dose- and time-dependent ROS changes in PC-3 and LNCaP cells after treated with α-TOS, TD-NPs, AD-NPs, and ATD-NPs were determined by flow cytometer. It can be observed that α-TOS, TD-NPs, and ATD-NPs could rapidly enhance intracellular ROS levels of both PC-3 cells (Fig. 4a, b) and LNCaP cells (Additional file 1: Fig. S10A and B). The ROS levels in PC-3 cells in TD-NPs and ATD-NPs group was 1.6- and 1.9-times than untreated cells even in the initial 1 h at α-TOS dose of 6 µg/mL, respectively, and after incubated 8 h it reached to 5.3- and 6.5-fold. Moreover, it can be found that even ATD-NPs and TD-NPs had the same content of α-TOS, the produce ROS ability of ATD-NPs was significant stronger than that of TD-NPs at each concentration point or time point in PC-3 cells. But this phenomenon was not found in LNCaP cells (Additional file 1: Fig. S10A and B), this may be contributed to DUP-1 mediate the actively cell targeting ability. The more α-TOS internalized into cells the higher the ROS level was. In addition, in compared with the control group, AD-NPs barely elevated the intracellular ROS level at each concentration and incubation time both in PC-3 cells and LNCaP cells. The fluorescence microscope images results revealed that ATD-NPs group exhibited stronger fluorescence intensity in PC-3 cells when compared to TD-NPs and α-TOS after incubated for 4 h, which was consistent with the quantitative results (Fig. 4d). These results indicated that the α-TOS could rapidly generate ROS in cancer cells.

Additionally, to demonstrate the mechanism of intracellular ROS production of α-TOS, the activity of mitochondrial respiratory complex II in PC-3 cells and LNCaP cells was evaluated. As present in Fig. 4c and Additional file 1: Fig. S10C, the activity of mitochondrial respiratory complex II was significantly inhibited by α-TOS, ATD-NPs, and TD-NPs in compared with AD-NPs, in which, AD-NPs had no obvious effect in the activity of mitochondrial respiratory complex II. As mentioned above, in PC-3 cells, the actively of mitochondrial respiratory complex II inhibition rate in ATD-NPs group was significantly higher than that of TD-NPs, because of more α-TOS internalized by cells. And this phenomenon not observed in LNCaP cells. The generated ROS of α-TOS could accelerate DOX and α-TOS release from ATD-NPs, which in turn induced ROS production, making a cycle of ROS regeneration with positive feedback, finally, improving deliver efficiently of ROS responsive drug delivery system.

### ROS triggered intracellular DOX release

**Fig. 5:**
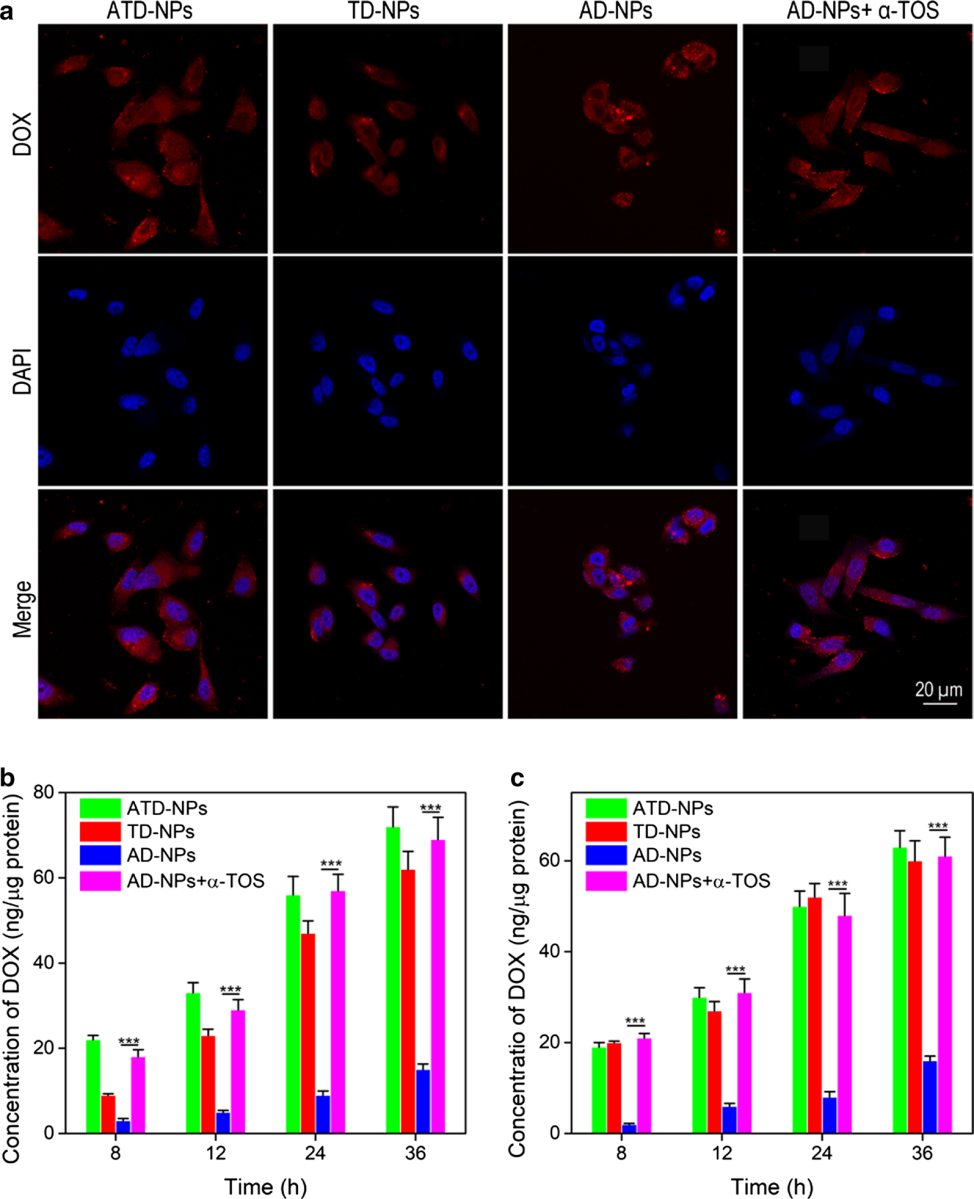
**a** CLSM images of PC-3 cells treated with ATD-NPs, TD-NPs, AD-NPs, and AD-NPs + α-TOS for 12 h. **b**, **c** Intracellular active DOX detected by HPLC in PC-3 cells (**b**) and LNCaP cells (**c**) after treated with ATD-NPs, TD-NPs, AD-NPs, and AD-NPs + α-TOS for different times. Data showed mean ± SD, *n* = 3. ***p < 0.001

Above-mentioned study showed the satisfying ROS generating ability of α-TOS, the ROS enhancement triggered by α-TOS released from the ATD-NPs induced DOX release in cancer cells was further confirmed by CLSM and HPLC. For the CLSM analysis, as shown in Fig. 5a, after incubated for 12 h, in the AD-NPs group, only slightly red fluorescence of DOX can be observed and almost accumulated in the cytoplasm. However, after added free α-TOS, stronger red fluorescence of DOX can be observed both at cytoplasm and nucleus. Similarly, the same phenomenon can be observed both in the TD-NPs and ATD-NPs group. These results could be explained by the fact that when DOX loaded into nanoparticles, which fluorescence was sharply decreased, and under low ROS conditions, the DOX cannot be released from AD-NPs. Therefore, in the AD-NPs group, only weak red fluorescence of DOX can be observed. Moreover, large particle size nanoparticles may prevent DOX entering the nucleus. Hence, in the AD-NPs group, almost no red fluorescence was observed in the nucleus. However, after treated with α-TOS, the cells generated a lot of ROS, which could trigger DOX release from nanoparticles, the fluorescence of DOX could be recovered, and then the free DOX quickly enterer nucleus. Thus, both free α-TOS + AD-NPs group and ATD-NPs group could observe stronger red fluorescence in the cytoplasm and nucleus.

To further confirm this phenomenon, the active DOX in the PC-3 cells and LNCaP cells were measured by HPLC. As shown in Fig. 5b, c, the intracellular concentration of active DOX in the AD-NPs treatment group only 15.1 ng/µg protein in PC-3 cells as well as 16.3 ng/µg protein in LNCaP cells after incubated for 36 h, but which was 72.2 ng/µg protein in PC-3 cells and 63.1 ng/µg protein in LNCaP cells in ATD-NPs treatment group. Moreover, due to the active-targeting ability of ATD-NPs, the intracellular active DOX in ATD-NPs group was higher than that of TD-NPs in PC-3 cells. This result further confirmed that the α-TOS could effectively generate ROS intracellular, and resulting in accelerating release of DOX.

### Evaluation of cytotoxicity in vitro of nanoparticles

**Fig. 6:**
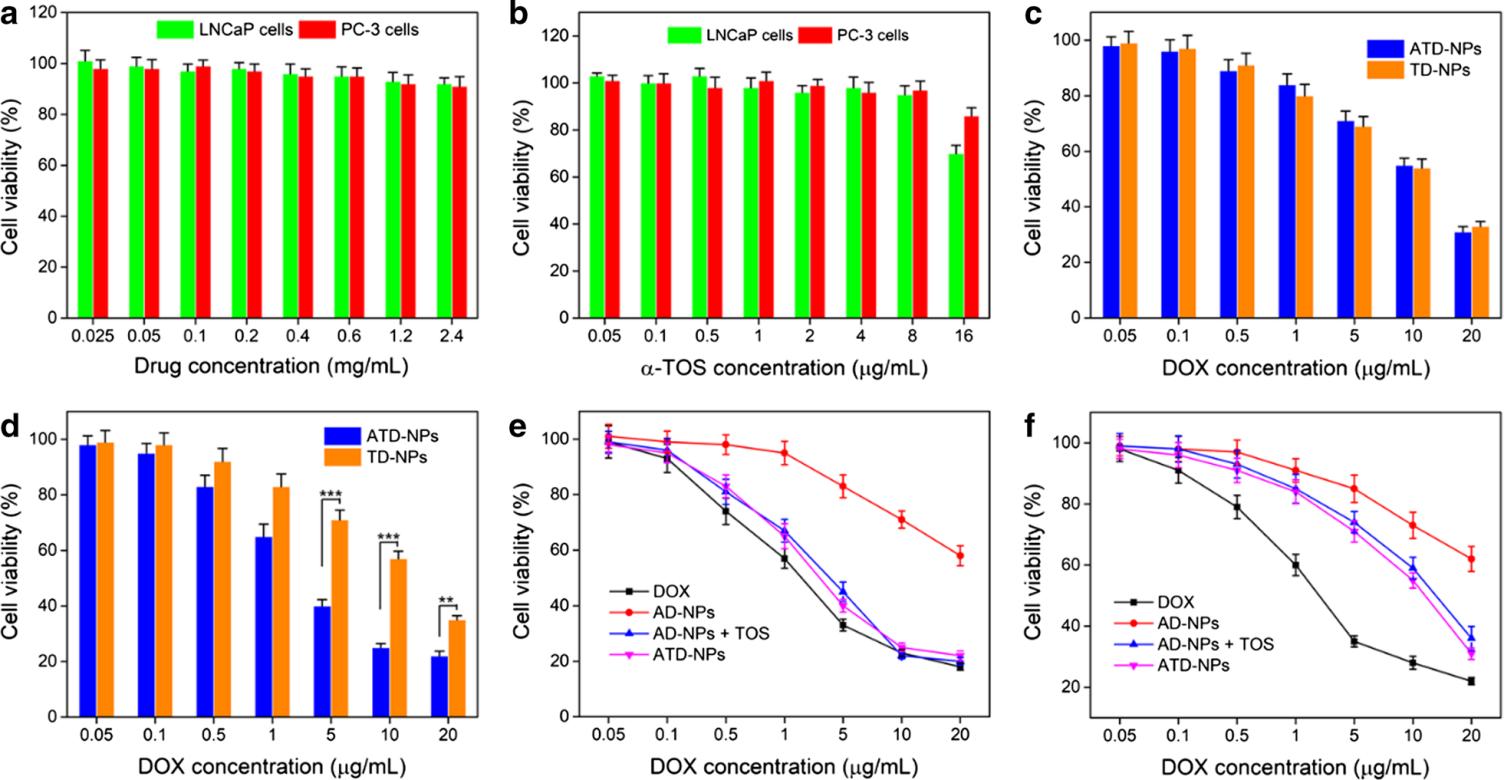
MTT assay of all drug formulation against PC-3 cells or LNCaP cells. **a** The relative cell viability of PC-3 cells and LNCaP cells after treated with PEG-*b*-P(LL-*g*-TK) for 48 h. **b** The relative cell viability of PC-3 cells and LNCaP cells after treated with α-TOS for 48 h. **c**, **d** The relative cell viability of LNCaP cells (**c**) and PC-3 cells (**d**) after treated with ATD-NPs and TD-NPs for 48 h. **e**, **f** The relative cell viability of PC-3 cells (**e**) and LNCaP cells (**f**) after treated with DOX, AD-NPs, AD-NPs + α-TOS, and ATD-NPs for 48 h. Data showed mean ± SD, *n* = 6. ***p < 0.001

The cytotoxicity of all drug formulations was evaluated by MTT assay. Firstly, the cytotoxicity of blank polymer was evaluated. As shown in Fig. 6a, after incubated with blank polymer for 48 h the cell viability of both LNCaP cells and PC-3 cells was higher than 90% even polymer concentration up to 2.4 mg/mL. This indicated that the PEG-*b*-P(LL-*g*-TK) we chose had a low toxicity and was suitable for drug delivery. Then, the cell viability of LNCaP cells and PC-3 cells after incubated with free α-TOS for 48 h was also both higher than 90%, this indicated that the free α-TOS has no cytotoxicity ranging 0.05 µg/mL from 16 µg/mL (Fig. 6b).

Additionally, to investigate the DUP-1 mediated active tumor targeting ability, the cytotoxicity of ATD-NPs or TD-NPs in PMSA (+) LNCaP cells and PMSA (−) PC-3 cells were measured. As provided in Fig. 6c, the cell viability of LNCaP cells after treated with ATD-NPs or TD-NPs had no obvious difference. IC_50_ value of ATD-NPs and TD-NPs was 11.4 µg/mL and 11.2 µg/mL (Additional file 1: Table S2), respectively. But the cell viability of ATD-NPs treated group on the PC-3 cells was significantly lower than that of TD-NPs treatment group (Fig. 6d) with the IC_50_ of ATD-NPs was 2.6 µg/mL (Additional file 1: Table S2), which was 3.9-fold lower than that of TD-NPs. This further indicated that DUP-1 could selectively kill PSMA (−) prostate cancer cells.

To further demonstrate the advantages of the ROS-triggered self-accelerating drug release nanosystem, the cell viability of PC-3 cells treated with ATD-NPs, AD-NPs, α-TOS + AD-NPs, and free DOX was analyzed by MTT assay. As presented in Fig. 6e, the free DOX exhibited the highest cytotoxicity, maybe at the in vitro conditions, DOX can be easily internalized by cells. The cell viability of AD-NPs in PC-3 cells was higher than 60%, even at the dosed as high as 10 µg/mL (equal to DOX), due to the limited DOX release caused by nonsufficient ROS during the incubation time. When the PC-3 cells were incubated with free α-TOS + AD-NPs, the cell viability sharply decreased, and was significantly lower than AD-NPs group. Similarly, the cell viability of ATD-NPs was also significantly lower than that of AD-NPs. Moreover, the IC50 of AD-NPs was 27.3 µg/mL (Additional file 1: Table S2), and it was 12.3- and 10.9-fold higher than that of free α-TOS + AD-NPs and ATD-NPs, respectively. This phenomenon was also found in LNCaP cells (Fig. 6f and Additional file 1: Table S2). These results demonstrated that the nanosystem we designed could actively target to the PMSA (−) prostate cancer and produce the cell toxicity selectively, particularly achieve enhanced chemotherapy compared to the no ROS amplification nanosystem (AD-NPs).

### In vivo circulation and biodistribution of nanoparticles

**Fig. 7:**
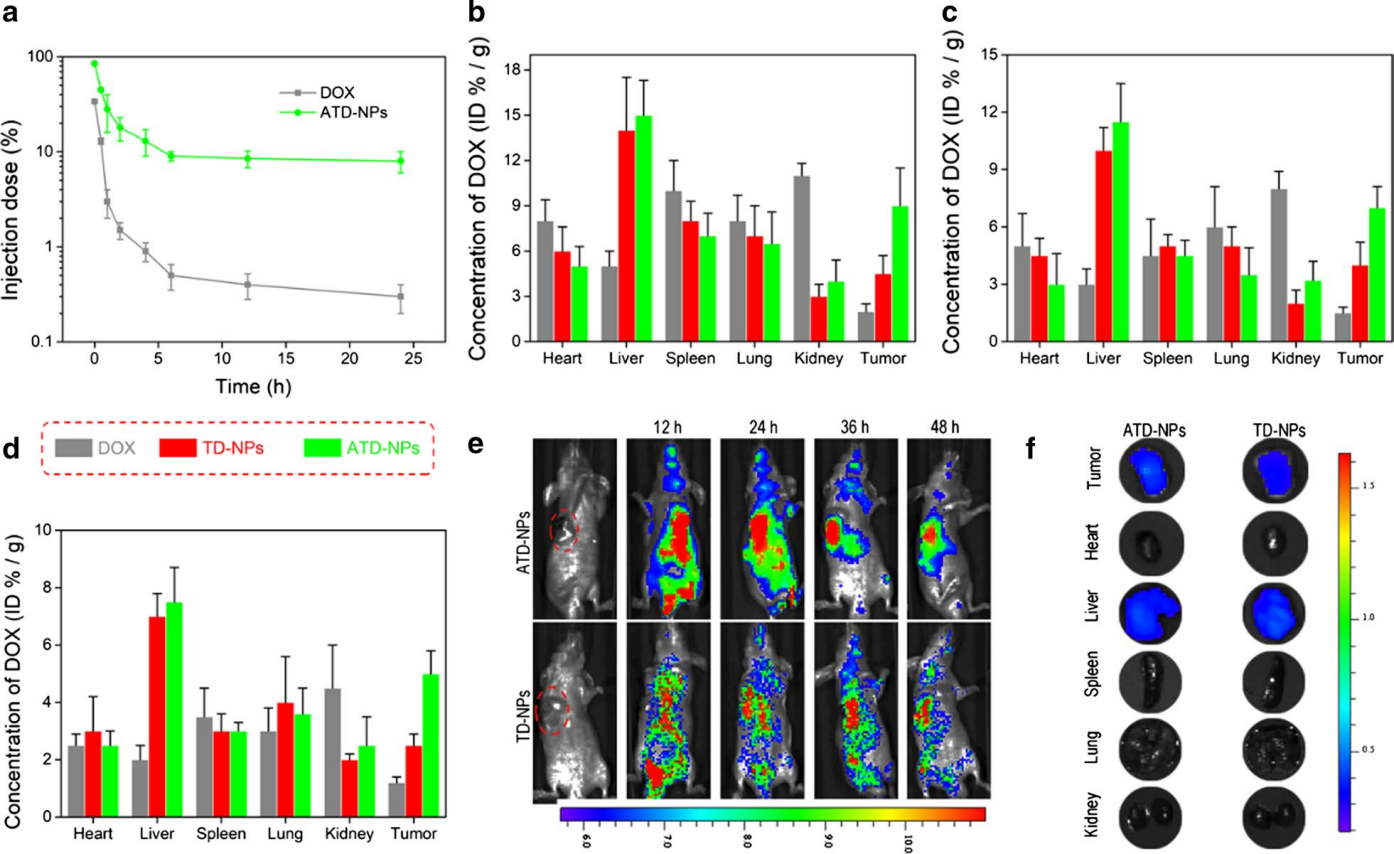
Blood circulation and biodistribution of different drug formulations. **a** Plasma DOX or DOX prodrug concentration as a function time after intravenous injection of free DOX and ATD-NPs. Data showed mean ± SD, *n* = 3. **b**, **d** Distribution of total DOX in organs or tissues at 4 h (**b**), 12 h (**c**), and 24 h. Data presented mean ± SD, *n* = 3. **d** After a single intravenous administration of free DOX, TD-NPs, and ATD-NPs, respectively. **e** In vivo fluorescence imaging of the PC-3 tumor bearing mice at 12 h, 24 h, 36 h, and 48 h after a single intravenous administration of cy5.5 loaded ATD-NPs and TD-NPs. **f** Ex vivo fluorescence images of isolated tissues at 48 h post-injection

Moreover, the in vivo circulation and biodistribution of free DOX and ATD-NPs were studied. After a single *i.v.* injection (equivalent DOX dose of 10 mg/kg), the concentration of total plasma DOX was determined. As shown in Fig. 7a, because ATD-NPs has the PEG shell protection, it displayed a much longer blood circulation than free DOX [4, 30]. The area under the concentration curve of ATD-NPs was 5.3-fold than free DOX, and at 12 h after injection, about 8% of the injected ATD-NPs remained in the plasma compared with only 0.7% of the free DOX.

In addition, the biodistribution of DOX, TD-NPs, and ATD-NPs in the major organs and tissues of PC-3-xenografted BALB/c mice was investigated. As illustrated in Fig. 7b–d, the high DOX concentration following injection of ATD-NPs and TD-NPs were found in the liver, spleen, and lung at 4 h, 12 h, and 24 h, which may be an indication of the reticuloendothelial system (RES) uptake. In addition, the DOX contents in the tumor following injection of ATD-NPs was 4.5-, 4.7, and 4.2-fold higher than that of free DOX at 4 h, 12 h, and 24 h, respectively; and the DOX content in ATD-NPs group was 2.0-, 1.8- and 2.0-times higher than that of TD-NPs after injected 4 h, 12 h, and 24 h, respectively. These results demonstrated that ATD-NPs had higher tumor targeting ability based on the passive (EPR effect) and active (DUP-1-mediated) targeting mechanisms.

To analyze the tumor-targeting ability of ATD-NPs, in vivo and ex vivo imaging experiments were employed to monitor ATD-NPs and TD-NPs time-dependent biodistribution in PC-3 tumor-bearing nude mice. Considering the imaging effect, we choose Cy5.5 as the fluorescence agent. As provided in Fig. 7e, f, the liver exhibited the strongest fluorescence signal in both in vivo and ex vivo images at 48 h after the administration. By contrast, the ATD-NPs group showed a higher fluorescence signal at tumor tissue than that in TD-NPs group. These results were consistent with the biodistribution data. The quantitative and qualitative analysis both indicated that the ATD-NPs could effectively accumulated in tumor site mediated by DUP-1, which may improve the therapy efficacy.

### In vivo antitumor efficacy

**Fig. 8:**
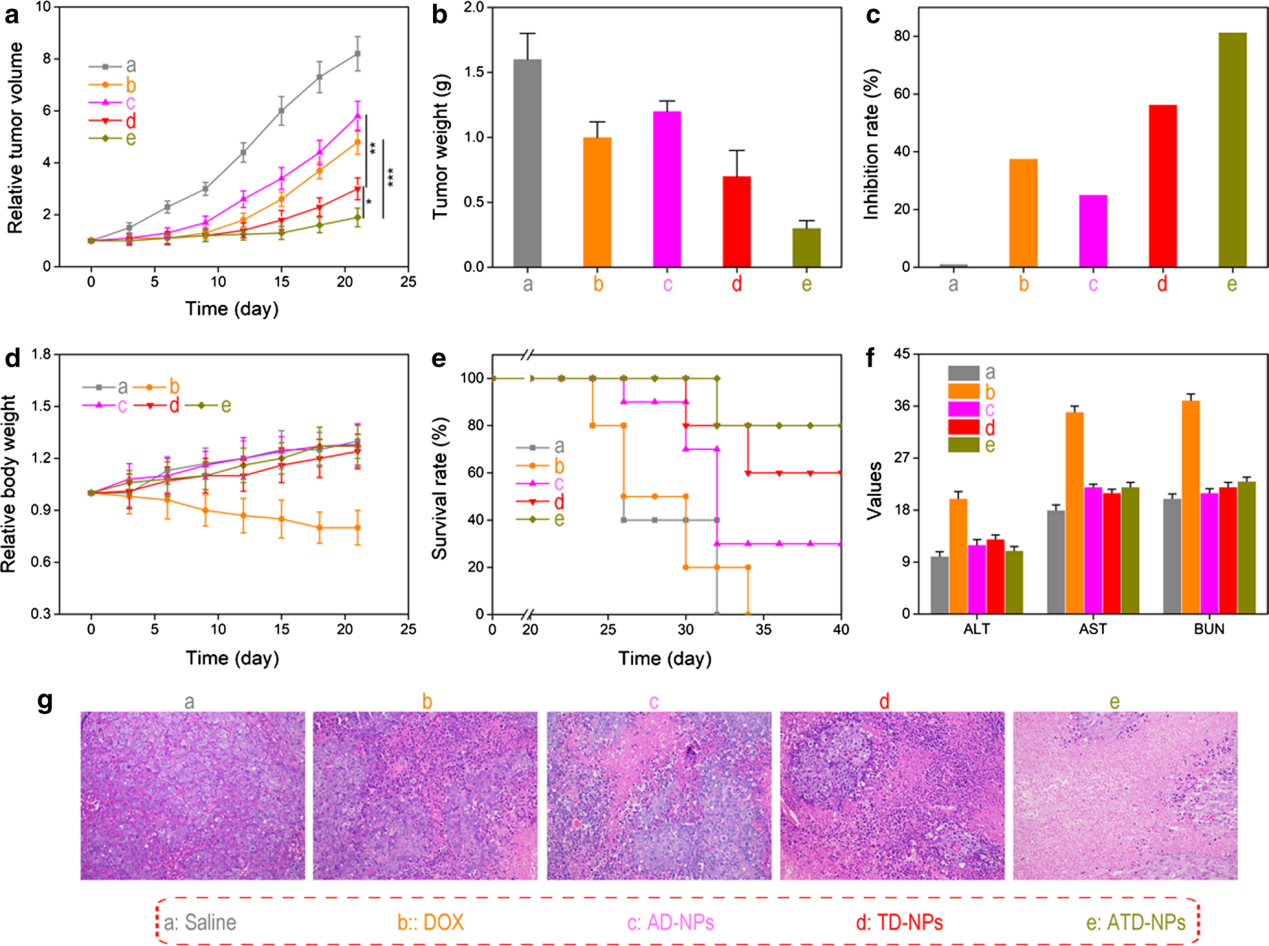
In vivo antitumor effects of different drug formulations. **a** Relative tumor volumes of PC-3 tumor-bearing mice after treatments with saline, DOX, AD-NPs, TD-NPs, and ATD-NPs (equivalent of 5 mg/kg DOX) for 21 days. **b** Extracted tumor weights. **c** Tumor inhibition rate. **d** Mice body weight. **e** Survival rates of mice after injected different formulations. **f** The alanine aminotransferase (ALT, 10 U/L), aspartate transaminase (AST, 10 U/L), and blood urea nitrogen (BUN, 100 µmol/L) in the serum of PC-3 tumor-bearing mice. **g** Images of H&E staining of tumor section acquired at 20× objective. Error bars showed as mean ± SD (n = 10), **p < 0.01, ***p < 0.001

The in vivo antitumor efficacy was then evaluated using human prostate cancer PC-3 tumor-bearing nude mice. After the tumor reached to a size of 100 mm^3^, tumor-bearing mice were randomly divided into five groups with ten mice in each group: PBS, DOX, AD-NPs, TD-NPs, and ATD-NPs, and then different formulations with equivalent doses of DOX (5 mg/kg) were given via tail *i.v.* injection at day 0, day 3, and day 6, respectively. The tumor size was measured every 3 day. Figure 8a showed the tumor volume as a function of time. Growth of the tumor was inhibited to a certain extent after the treatment with all drugs compared with the PBS control group. The anticancer efficacy of the AD-NPs group was very limited may be because the DOX cannot be effectively released from AD-NPs under intracellular ROS condition. As expected, the tumor growth in the group injected with ATD-NPs was noticeably inhibited, because of the advantages of active tumor targeted and ROS amplification function. The tumor weight of the excised tumor (Fig. 8b) agreed well with that measured in living mice (Fig. 8a). Moreover, the tumor inhibition rate of ATD-NPs was 81%, which was significantly higher than that of DOX (38%), AD-NPs (25%), and TD-NPs (56%) (Fig. 8c). Additionally, morphology of tumor cells in tumor tissues and therapeutic efficiency was evaluated by hematoxylin and eosin (H&E) staining assay. As exhibited in Fig. 8g, amount and compact spherical cells can be found in PBS group, however, in all drug treated groups, the cells in ATD-NPs treated group showed serious damage and many cells were dead. This result was consistent with the above results.

Meanwhile, in contrast to the free DOX group, which showed severe body weight loss during the treatment, the all nanoparticles group showed a steady increase in body weight (Fig. 8d). The body weight loss of DOX group would be contributed the side effect of DOX, and the cardiotoxicity is the main side effect of DOX.2,35 To confirm this phenomenon, a histological study was conducted by staining heart tissue sections with H&E. As shown in Additional file 1: Fig. S11, while DOX induced apparent necrosis in myocardial cells, the cardiac muscle fibers in the AD-NPs, TD-NPs, and ATD-NPs groups appeared normal, suggesting that the nanosystem could avoid the severe DOX cardiotoxicity. The survival rate of tumor-bearing mice showed that 60% mice were surviving after administration ATD-NPs 60th days (Fig. 8e). The survival of AD-NPs group and TD-NPs group at the end experiment time was 30% and 60%. Respectively. However, all mice died at 32th days and 34 days post administration of saline and DOX, respectively. In addition, the level of alanine aminotransferase (ALT), aspartate aminotransferase (AST) and blood urea nitrogen (BUN) also suggested that ATD-NPs had no significantly toxicity to the liver and kidney (Fig. 8f). These results demonstrated that the DUP-1 mediated tumor-active targeting and ROS-responsive drug delivery system with self-accelerated drug release could effectively improve therapeutic effect and reduce side effects.

## Conclusion

In summary, we have successfully developed a PMSA (−) prostate cancer actively targeted and self-amplification drug release system for enhanced tumor chemotherapy. The in vitro and in vivo experiments showed that the ATD-NPs not only can selectively targeted PSMA (−) tumor via DUP-1 mediated tumor active targeting, but also efficiently increased ROS level in cancer cell to achieve complete drug release. These two key features of ATD-NPs resulting in obviously enhance the tumor therapeutic efficacy and reduce the systematic toxicity of chemotherapeutics, such as DOX. The described technology unifies the tumor actively targets, self-amplified drug release, and excellent biocompatibility into one formulation, are promising for cancer treatment.

## Additional file

**Additional file 1.** Supporting Information.
