# Supplementary material for: A ROS-responsive polymeric prodrug nanosystem with self-amplified drug release for PSMA (−) prostate cancer specific therapy
Source: J Nanobiotechnology. 2019 Aug 26;17:91. doi: 10.1186/s12951-019-0521-z (PMC6709549; doi:10.1186/s12951-019-0521-z)
Supplement: Supplementary file 1 — Additional file 1. Supporting Information. [file 12951_2019_521_MOESM1_ESM.docx]

**A ROS-responsive polymeric prodrug nanosystem with self-amplified drug release for PSMA (-) prostate cancer specific therapy**

Yifan Wang^1#^, Yanqiu Zhang^2#^, Zhengxing Ru^3^, Wei Song^4^, Lin Chen^2^, Hao Ma^2^, Lizhu Sun^2*^

1. Department of Oncology, Yancheng City No.1 People’s Hospital, YanCheng, 224005, China

2. Department of Oncology, Shuyang Hospital Affiliated to Xuzhou Medical University, Shuyang People's Hospital, Suqian, 223600, China

3. Department of Oncology, Nanjing First Hospital, Affiliated to Nanjing Medical University, Nanjing, 210015, China

4. Department of Oncology, Nanjing Hospital of T.C.M, Affiliated to Nanjing University of Traditional Chinese Medicine, Nanjing, 210001, China

**Corresponding author:**

Lizhu Sun, Department of Oncology, Shuyang Hospital Affiliated to Xuzhou Medical University, Shuyang People's Hospital, Suqian, 223600, China

^*^ E-mail: [dorslz@163.com](mailto:dorslz@163.com),

**^#^ These authors contributed equally to this manuscript.**

**1. Materials**

DOX·HCl was purchased from Beijing Huafeng United Technology Co., Ltd (Beijing, China). D-α-tocopheryl succinate (α-TOS) was purchased from Shanghai Yuanye Bio-Technology Co., Ltd (Shanghai, China). DSPE-PEG(5000) Maleimide (Mal-PEG-DSPE), DSPE-PEG(5000) methoxyl (mPEG-DSPE), N-hydroxysuccinimide (NHS), 1-(3-dimethylaminopropyl)-3-ethylcarbodiimide hydrochloride (EDC), and 3-mercaptopropionic acid were purchased from Aladdin Reagent Co. Ltd. (Shanghai, China). *N*^ε^-Benzyloxycarbonyl-L-lysine-*N*-carboxyanhydride (Lys-NCA) was purchased from Jiangsu Beta pharma Co., Ltd (Suzhou, China). Amino-terminated methoxyl poly(ethylene glycol) (PEG-NH_2_, Mw = 5000) was obtained from JenKem Technology Co., Ltd (Beijing, China). The C-terminus cysteine modified DUP-1 peptide (CFRPNRAQDYNTN) was synthesized by Sangon Biotech (shanghai) Co., Ltd. Dichlorofluorescindiacetate (DCFA-DA), BCA kit, 3-(4,5-Dimethylthiazol-2-yl)-2,5-diphenyl tetrazolium bromide (MTT) and DAPI were purchased from Beyotime Institute of Biotechnology (Shanghai, China). Mitochondrial Complex II Activity Assay Kit was obtained from Cayman Chemical (USA).

**2. Cells and animals**

The human prostate cancer cell line PSMA (+) LNCaP and PSMA (-) PC-3 were obtained from Institute of Biochemistry and Cell Biology, Shanghai Institutes for Biological Sciences, Chinese Academy of Sciences (Shanghai, China) and cultured in RPMI 1640 culture medium, containing 10% (v/v) feral bovine serum, 100 IU/mL penicillin, and 100 µg/mL streptomycin at 37 ℃ in a humidified 5% CO_2_ atmosphere.

BALB/c nude mice (male, 4 - 6 weeks, 20 ± 2 g) and ICR mice were purchased from the Vital River Laboratory Animal Technology Co., Ltd. (Beijing, People’s Republic of China). All animals received care in compliance with the guidelines outlined in the Guide for the Care and Use of Laboratory Animals and all procedures were approved by The Shuyang Hospital Affiliated to Xuzhou Medical University Care and Use Committee.

**3. Characterization**

Nuclear magnetic resonance (NMR) spectra were recorded on a Bruker AVANCE III spectrometer at 300 MHz with deuterated dimethyl sulfoxide (DMSO-*d6*), CD_3_OD, or D_2_O as the solvent. Molecular weight distributions (polydispersity index, PDI = Mw/Mn) of the copolymer were determined by gel permeation chromatography (GPC) using a Waters GPC system[^1^](#_ENREF_1) (Waters Styragel HT6E column, with OPTILAB DSP interferometric refractometer as the detector). DMF was used as the eluent at a flow rate of 1.0 ml min^−1^ at 40 °C. Poly(ethylene glycol) with different molecular weights were used to generate the calibration curves. The size, size distribution and the zeta potential of nanoparticles in aqueous solution were determined by dynamic light scattering (DLS, Zs90, Malvern Instruments, Malvern, UK). The morphology of particles was investigated using transmission electronic microscopy (TEM, Hitachi Ltd, Tokyo, Japan). The mass studies were performed on a QTRAP 5500 Tripe Quad mass spectrometer (AB SCIEX, USA) by using the electrospray ion source.

**4. Synthesis of DUP-1 conjugated Mal-PEG-DSPE (DUP-PEG-DSPE)**

DUP-PEG-DSPE was synthesized through Michael addition reaction between DUP-1 and Mal-PEG-DSPE. Typically, Mal-PEG-DSPE (5 mmol) and DUP-1 (7 mmol) were dissolved in DMF (2 mL) or PBS (2 mL), respectively, and followed by adding 20 mL of PBS under stirring with a nitrogen atmosphere at room temperature. After stirred for 4 h, the reaction mixture was purified via dialysis (molecular weight cutoff (MWCO) = 3,500 Da) against distilled water for 24 h, and then lyophilized to obtain the product DUP-PEG-DSPE.

**5. Synthesis of ROS-sensitive thioketal linker**

The thioketal linker was prepared according to the previous report.[^2^](#_ENREF_2) Briefly, anhydrous acetone (6.8 g, 115.6 mM) and anhydrous 3-mercaptopropinonic (6.0 g, 56.6 mM) were dissolved with dry hydrogen chloride and the mixture were stirred at room temperature for 6 h. Then, the reaction was quenched by placing the mixture in an ice-salt mixture bath until the crystallization was completed. Finally, the solution was filtered, washed with abundant hexane and cold water. The product was obtained after drying under vacuum.

**6. Synthesis of PEG-*b*-PLL**

Methoxy poly(ethylene glycol)-*b*-poly(L-lysine) (PEG-*b*-PLL) copolymer was synthesized by ring opening polymerization of *N*^ε^- benzyloxycarbonyl-L-lysine-*N*-carboxyanhydride (Lys-NCA) using amino-terminated methoxyl poly(ethylene glycol) (PEG-NH_2_) as a macroinitiator according to reference.[^3^](#_ENREF_3) Briefly, Lys-NCA (3.1 g, 10 mM) was dissolved in 30 mL of dry DMF. Then, PEG-NH_2_ (2.5 g, 0.5 mM) was dissolved in 40 mL of dry DMF as the macroinitiator and added to Lys-NCA solution *via* a syringe under dry argon. The reaction was maintained at 40 ℃ under a dry argon atmosphere for 3 days. The mixture was concentrated under vacuum. The methoxy poly(ethylene glycol)-*b*-poly(*N*^ε^-benzyloxycarbonyl-L-lysine) (PEG-*b*-PLLZ) copolymer was obtained by repeated precipitation from DMF into excess ice-cold diethyl ether.

Subsequently, PEG-*b*-PLLZ (5.0 g) was dissolved in 50 mL of trifluoroacetic acid (TFA) at 0 ℃, and then 15 mL HBr/acetic acid (33%) was added at 25 ℃ under quick stirring and reacted for 1 h to remove the protecting groups. After that, the reaction mixture was precipitated in excessive cold diethyl ether. The product was further purified by dialyzed (MWCO = 3,500 Da) against distilled water. A white solid was obtained by lyophilization.

**7. Synthesis of ROS-sensitive DOX prodrug**

The ROS-responsive DOX prodrug: PEG-*b*-P((LL-*g*-TK)-(LL-*g*-TK-DOX)) (named as P(L-TK-DOX)) was prepared by two steps. Firstly, TK was conjugated to the side amino of PEG-*b*-PLL through amidation reaction to obtain the PEG-*b*-P(LL-*g*-TK); then, DOX was conjugated to the side of PEG-*b*-P(LL-*g*-TK) also by amidation reaction to obtain the final product P(L-TK-DOX). In brief, TK (108.3 mg, 0.43 mM), EDC (99.0 mg, 0.52 mM), and NSH (74.2 mg, 0.65 mM) were dissolved in 40 mL dry *N, N*-Dimethylformamide (DMF) and the mixture was stirred at room temperature for 2 h to active the carboxyl of TK. After that, PEG-*b*-PLL (146.0 mg, 0.02 mM) and appropriate amount triethylamine (TEA) were dissolved in 30 mL dry DMF and added dropwise to the above reaction solution at vigorously stirring. The reaction mixture was stirred for 24 h at room temperature under a dry argon atmosphere and then the extra solution was evaporated under reduced pressure. The product was further purified by dialyzed (MWCO = 3,500 Da) against distilled water. The PEG-*b*-P(LL-*g*-TK) was obtained by lyophilization.

The P(L-TK-DOX) was obtained through the amidation reaction between DOX and PEG-*b*-P(LL-*g*-TK). Briefly, PEG-*b*-P(LL-*g*-TK) (108.0 mg, 0.01 mM), EDC (3.9 mg, 0.2 mM) and NHS (4.6 mg, 0.04 mM) were dissolved in 20 mL of dimethyl sulfoxide (DMSO) and the reaction mixture was maintained under stirring for 1 h at room temperature. Then, DOX·HCl (173.3 mg, 0.3 mM) and proper amount triethylamine (TEA) were dissolved in 10 mL of DMSO, and then added into the above-mentioned solution drop by drop under vigorously stirring, the reaction was maintained at room temperature for 48 h under a dry argon atmosphere. The product was purified by dialyzed (MWCO = 3500 Da) against DMSO to remove unreacted DOX, and the against distilled water to remove DMSO. The P(L-TK-DOX) was obtained by lyophilization.

**8. Stability test of ATD-NPs in FBS**

The stability of ATD-NPs in serum was investigated by incubating micelle solution in PBS (pH 7.4) at 37 ℃ with or without 10% FBS. The average size of ATD-NPs micelle was measured at intervals time using DLS.

**9. Drug release in different ROS conditions**

Drug release profile were conducted in four solutions with different concentrations of H_2_O_2_: PBS (pH 7.4), PBS (pH 7.4) with 20 nM H_2_O_2_, PBS (pH 7.4) with 0.1 mM H_2_O_2_, and PBS (pH 7.4) with 1 mM H_2_O_2_ at 37 ℃. 2 mg of ATD-NPs (content about 0.4 mg DOX) was suspended in the above three buffer solution (2 mL), respectively. Subsequently, the suspension was transferred to a dialysis tube (MWCO: 3500 Da) and immersed in a threaded bottle with 28 mL the above PBS buffer solutions and contented 2% Tween 80. At certain intervals, a 500 µL aliquot of the solution outside the bag was withdrawn for HPLC analysis and added the same volume fresh release buffer into the threaded bottle at the same time. The HPLC condition of α-TOS was listed at above. The HPLC condition of DOX was listed as following: the HPLC analysis was performed using a C18 phase column eluted with acetonitrile: methanol with 0.03% trifluoroacetic acid (50/50, v/v) at a flow rate of 1 mL/min and the column effluent was monitored by fluorescence detector set at 470/560 nm.[^4^](#_ENREF_4)

**10. Cell uptake of targeted delivery system of ATD-NPs**

To study the DUP-1 mediated PSMA (-) prostate cancer active targeting drug delivery, the PMSA (-) PC-3 cells were seeded at 1 × 10^4^ cells per well in six-well plates 48 h before the cellular uptake assay. Then, the cells were treated with targeted ATD-NPs nanoparticles and non-targeted TD-NPs nanoparticles at the concentration of DOX at 10 µg/mL. After incubated for 1 h or 2 h, the cells were fixed with 4% paraformaldehyde solution, stained by DAPI, washed with pre-cold PBS, and then observed by fluorescence microscope (Imager A1, Zeiss, Germany).

To further quantitatively analyze the cellular uptake, PC-3 cells were seeded onto six-well plates and incubated for 48 h. Then cells were treated with ATD-NPs or TD-NPs nanoparticles for different times. After that PC-3 cells were washed, trypsin digested, collected and analyzed in the FL2 channel on BD FACS Calibur flow cytometer equipped with a 488 nm argon laser used to scan DOX fluorescence. Moreover, intracellular concentration of DOX in PSMA (+) LNCaP cells were also quantitatively analyzed by flow cytometer at the same conditions.

**11. *In vitro* cytotoxicity study**

The MTT assay was employed to evaluate the cytotoxicity of all drug forms. In brief, PC-3 cells and LNCaP cells were seeded in the 96-well plates at a density of 5 × 10^3^ cells per well for 24 h. Then, the cells were treated with DOX, α-TOS, AD-NPs, AD-NPs + α-TOS, TD-NPs, or ATD-NPs at different concentration. After incubating for 48 h, 20 µL of MTT (5 mg/mL) was added to each well and incubated for another 4 h. After that, cells were dissolved in 200 µL of DMSO, the absorbance was detected at 490 nm using a microplate reader (Thermo Multiclan MK3; Thermo Fisher Scientific, USA).

In addition, the cytotoxicity of PEG-*b*-P(LL-*g*-TK) polymer for PC-3 cells and LNCaP cells were also evaluated by MTT assay at different concentrations.

**12. Hemolysis assay**

Freshly mice blood was diluted by PBS (pH 7.4), and red blood cells (RBCs) were collected by centrifugation. The RBCs were further diluted by PBS. Then, the RBCs suspension was added to the ATD-NPs, TD-NPs, and AD-NPs micelles solution, the concentration of all micelles is ranging 0.01 from 5 mg/mL. The mixtures were maintained at 37 ℃ for 2 h after a gently shaking. After that, the mixtures were centrifuged, and the absorbency (Ab) of the supernatant of each sample was detected by a microplate reader at 540 nm. PBS and TritonX-100 (2%) were used as negative and positive controls, respectively. The hemolysis ratio (HR) of RBCs was calculated according to the following formula:

HR (%) = $\frac{Asample-Anegative control}{Apostive control-A negative control}\times100\%$.

All hemolysis experiments were carried out in triplicate.

**13. *In vivo* imaging of mouse with xenograft tumor**

The prostate cancer tumor model was established by subcutaneous injection of 7 × 10^6^ PC-3 cells into the right side back of male nude mice. After two weeks, the Cy5.5 labeled ATD-NPs (DLC: 1.8%) or TD-NPs (DLC: 2.3) nanoparticles was intravenously injected *via* the tail vein. At 12, 24, 36 and 48 h post injection, the mice were imaged on IVIS Lumina imaging system (Caliper, USA) and the excitation and emission wavelength was set as 675 nm and 694 nm, respectively. Thereafter, the mice were euthanized at 36 h post injection, tumors and the major organs, such as heart, live, spleen, lung, and kidney, and subjected to *ex vivo* fluorescence imaging.





**Scheme S1** Synthesis route of TK (A), DUP-PEG-DSPE (B), and P(L-TK-DOX) (C).


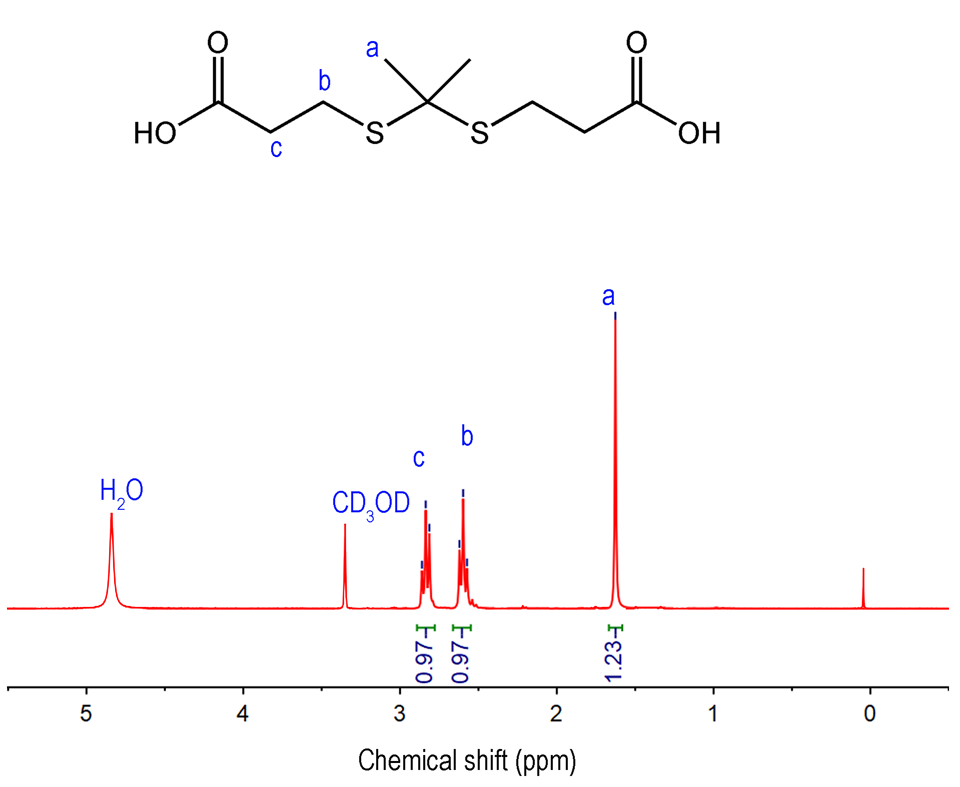


**Fig. S1** ^1^H NMR spectrum of TK in CD_3_OD.


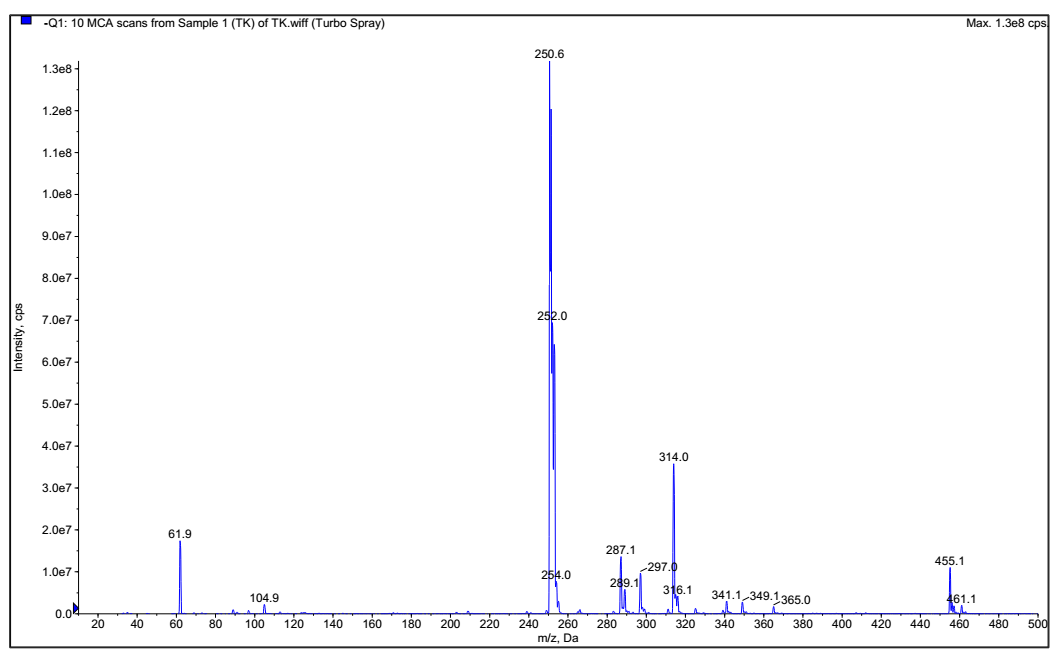


**Fig. S2** Electrospray ionization mass spectrum of TK.


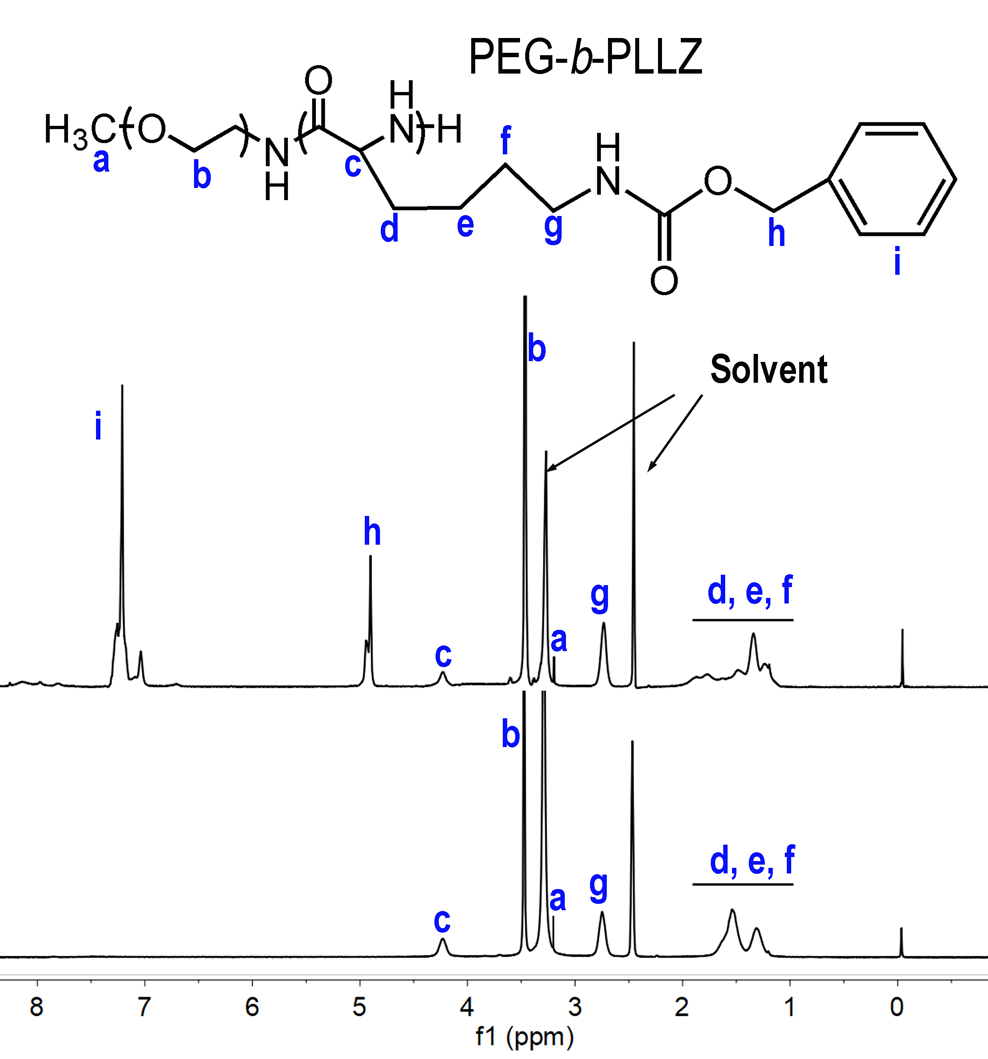


**Fig. S3** ^1^H NMR spectrum of PEG-b-PLL (above) and PEG-b-PLL (below) in DMSO-*d6*.


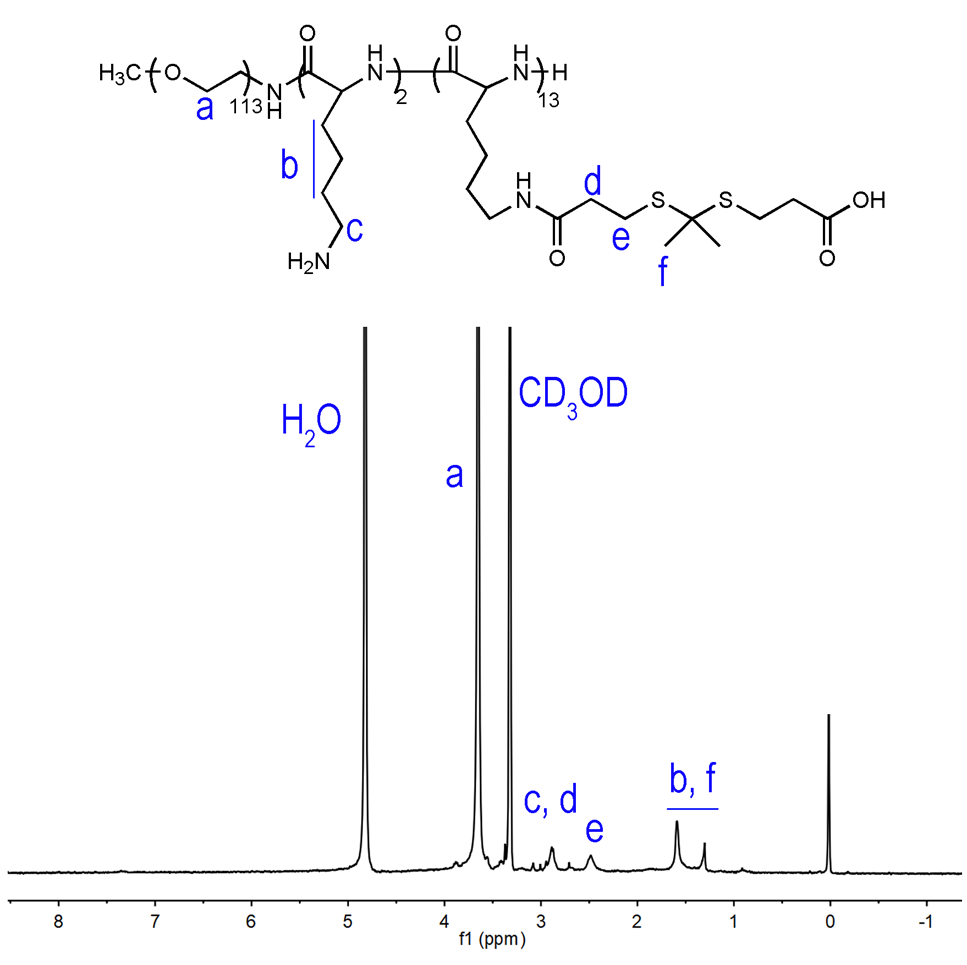


**Fig. S4** ^1^H NMR spectrum of PEG-P(LL-g-TK) in CD_3_OD.


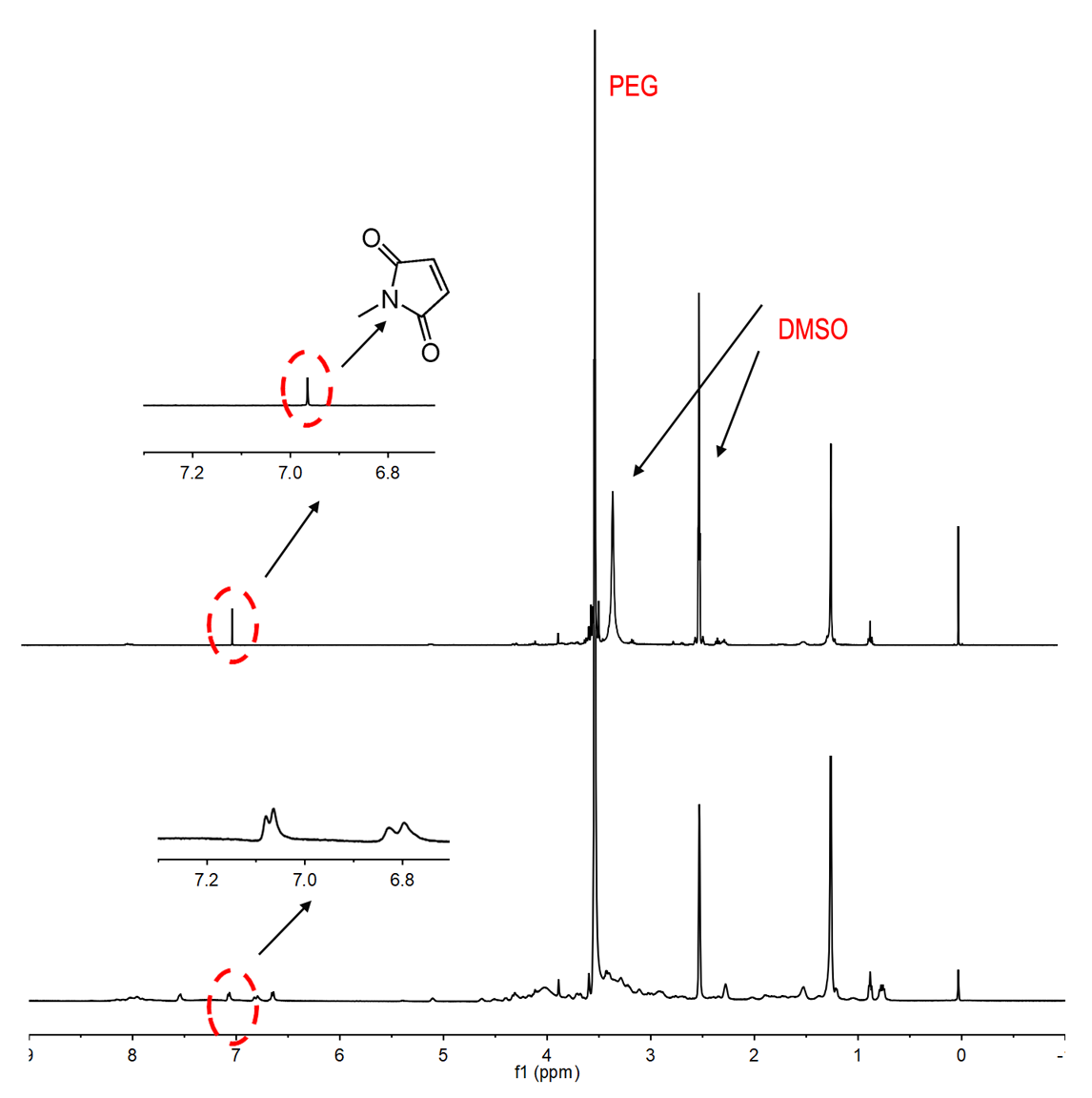


**Fig. S5** ^1^H NMR spectrum of Mal-PEG-DSPE (above) and DUP-PEG-DSPE (below) in DMSO-d6.





**Fig. S6** Critical aggregation concentration of ATD-NPs, TD-NPs and AD-NPs.


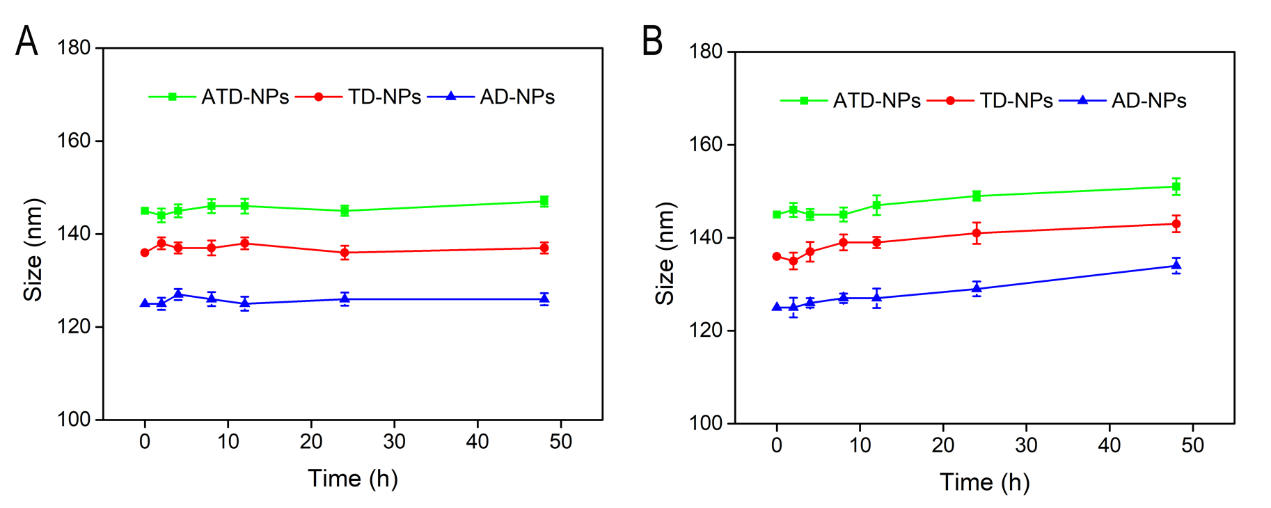


**Fig. S7** Stability of ATD-NPs, TD-NPs, and AD-NPs in PBS (A) and PBS contained 10% FBS (B).





**Fig. S8** Hemolysis rate of ATD-NPS, TD-NPs, and AD-NPs at the rang of 0.01 to 5 mg/mL.


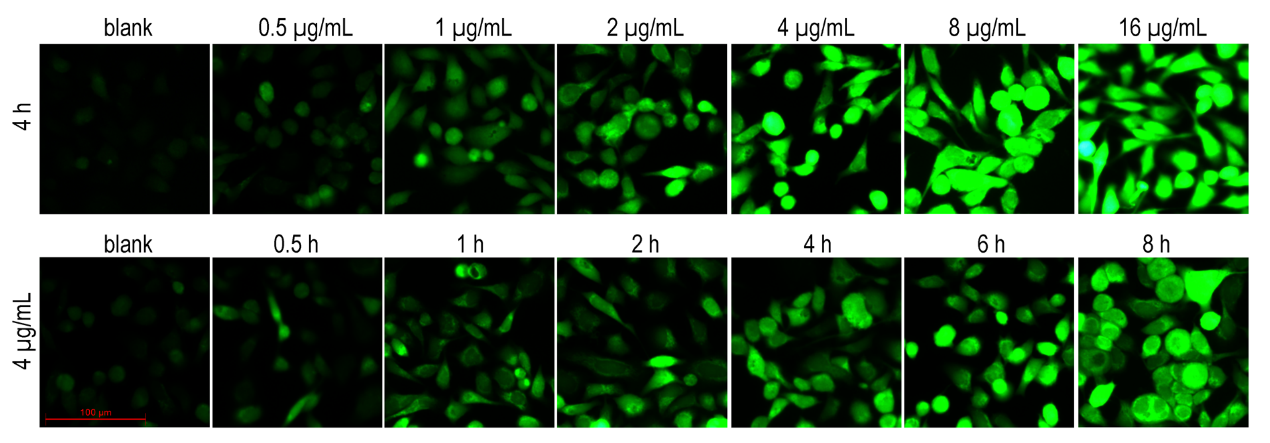


**Fig. S9** Evaluation of the ROS regeneration ability of α-TOS in vitro. Fluorescence microscope image of PC-3 cells treated with different concentration of α-TOS for 4 h (A) and 4 µg/mL of α-TOS for different time.


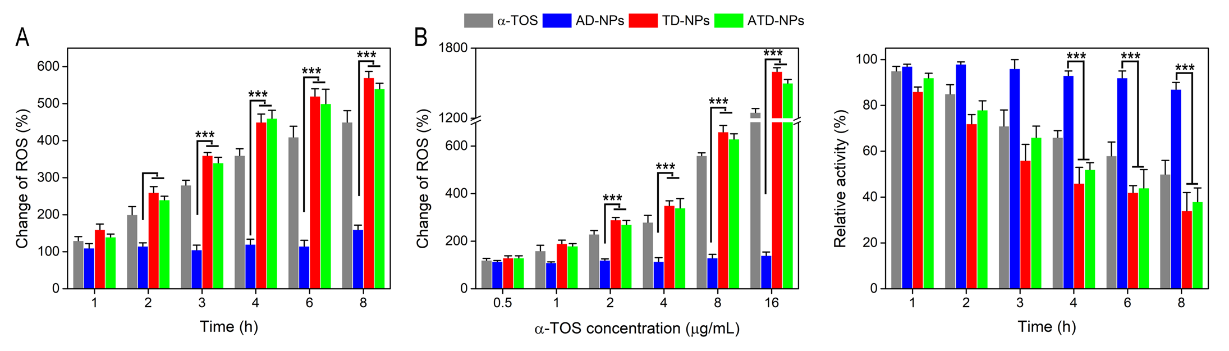


**Fig. S10** ROS changes in LNCaP cells after treated with α-TOS, TD-NPs, AD-NPs, and ATD-NPs for different incubation times (A) and different concentration (B). For (A), the concentration of α-TOS in all group was fixed at 6 µg/mL; for (B), all group was treated for 4 h. (C) The relative activity of mitochondrial respiratory complex II in LNCaP cells after incubated with α-TOS, TD-NPs, AD-NPs, and ATD-NPs for different time. Data showed as mean ± SD, *n* = 3. ** *p* < 0.01, *** *p* < 0.001.


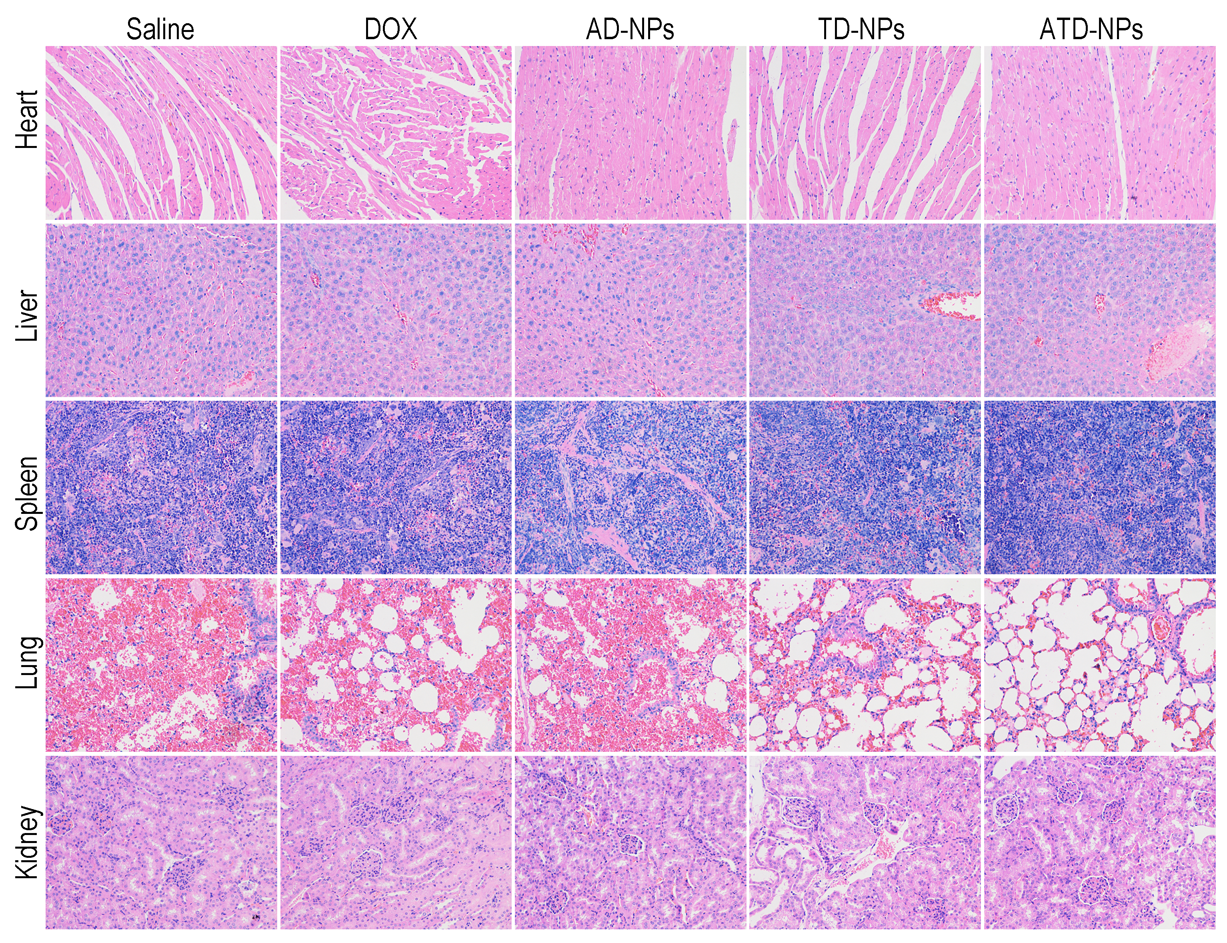


**Fig. S11** Hematoxylin-eosin staining (H&E) analysis of major organs (heart, liver, spleen, lung, and kidney) of mice after treated with saline, DOX, ATD-NPs, TD-NPs, and AD-NPs, respectively.

| **Table. S1** The component of AD-NPs, TD-NPs, and ATD-NPs. | | | |
| --- | --- | --- | --- |
| Element | AD-NPs | TD-NPs | ATD-NPs |
| P(L-TK-DOX) | + | + | + |
| DUP-PEG-DSPE | + | - | + |
| PEG-DSPE | - | + | - |
| α-TOS | - | + | + |

+: exhibited including; -: mean not including.

| **Table. S2** IC50 value (µg/mL) in different formulations against PC-3 and LNCaP. | | | | | |
| --- | --- | --- | --- | --- | --- |
|  | DOX | AD-NPs | AD-NPs + α-TOS | TD-NPs | ATD-NPs |
| PC-3 | 1.1 | 28.3 | 2.3 | 10.2 | 2.6 |
| LNCaP | 1.3 | 29.8 | 11.3 | 11.2 | 11.4 |

1. Lv, S.; Li, M.; Tang, Z.; Song, W.; Sun, H.; Liu, H.; Chen, X. *Acta Biomaterialia* **2013,** 9, (12), 9330-9342.

2. Chen, W. H.; Luo, G. F.; Qiu, W. X.; Lei, Q.; Hong, S.; Wang, S. B.; Zheng, D. W.; Zhu, C. H.; Zeng, X.; Feng, J.; Cheng, S. X.; Zhang, X. Z. *Small* **2016,** 12, (6), 733-44.

3. Lv, S.; Tang, Z.; Zhang, D.; Song, W.; Li, M.; Lin, J.; Liu, H.; Chen, X. *Journal of controlled release : official journal of the Controlled Release Society* **2014,** 194, 220-7.

4. Wang, F.; Wang, Y. C.; Dou, S.; Xiong, M. H.; Sun, T. M.; Wang, J. *ACS nano* **2011,** 5, (5), 3679-3692.
